# Roles of the *14-3-3* gene family in cotton flowering

**DOI:** 10.1186/s12870-021-02923-9

**Published:** 2021-03-31

**Authors:** Na Sang, Hui Liu, Bin Ma, Xianzhong Huang, Lu Zhuo, Yuqiang Sun

**Affiliations:** 1 The Key Laboratory of Oasis Eco-Agriculture, College of Agriculture, Shihezi University, Shihezi, 832000 China; 2 Special Plant Genomics Laboratory, College of Life Sciences, Shihezi University, Shihezi, 832000 China; 3 Center for Crop Biotechnology, College of Agriculture, Anhui Science and Technology University, Fengyang, 233100 China; 4 Plant Genomics & Molecular Improvement of Colored Fiber Laboratory, College of Life Sciences and Medicine, Zhejiang Sci-Tech University, Hangzhou, 310016 Zhejiang China

**Keywords:** 14-3-3, GENERAL REGULATORY FACTOR, Florigen activation complex, Flowering, Cotton

## Abstract

**Background:**

In plants, 14-3-3 proteins, also called GENERAL REGULATORY FACTORs (GRFs), encoded by a large multigene family, are involved in protein–protein interactions and play crucial roles in various physiological processes. No genome-wide analysis of the *GRF* gene family has been performed in cotton, and their functions in flowering are largely unknown.

**Results:**

In this study, 17, 17, 31, and 17 *GRF* genes were identified in *Gossypium herbaceum*, *G. arboreum*, *G. hirsutum*, and *G. raimondii*, respectively, by genome-wide analyses and were designated as *GheGRF*s, *GaGRF*s, *GhGRF*s, and *GrGRFs*, respectively. A phylogenetic analysis revealed that these proteins were divided into ε and non-ε groups. Gene structural, motif composition, synteny, and duplicated gene analyses of the identified *GRF* genes provided insights into the evolution of this family in cotton. *GhGRF* genes exhibited diverse expression patterns in different tissues. Yeast two-hybrid and bimolecular fluorescence complementation assays showed that the GhGRFs interacted with the cotton FLOWERING LOCUS T homologue GhFT in the cytoplasm and nucleus, while they interacted with the basic leucine zipper transcription factor GhFD only in the nucleus. Virus-induced gene silencing in *G. hirsutum* and transgenic studies in *Arabidopsis* demonstrated that *GhGRF3/6/9/15* repressed flowering and that *GhGRF14* promoted flowering.

**Conclusions:**

Here, 82 *GRF* genes were identified in cotton, and their gene and protein features, classification, evolution, and expression patterns were comprehensively and systematically investigated. The GhGRF3/6/9/15 interacted with GhFT and GhFD to form florigen activation complexs that inhibited flowering. However, GhGRF14 interacted with GhFT and GhFD to form florigen activation complex that promoted flowering. The results provide a foundation for further studies on the regulatory mechanisms of flowering.

**Supplementary Information:**

The online version contains supplementary material available at 10.1186/s12870-021-02923-9.

## Background

The 14-3-3 proteins are highly conserved in virtually every eukaryotic organisms and tissue [1]. Among plants, *14-3-3* genes have been studied most thoroughly in *Arabidopsis thaliana*, in which the *14-3-3* genes are designated either as G-box Factor 14-3-3 homologs (*GF14*s) or General regulatory factors (*GRFs*) [2–4]. The 14-3-3 proteins exist in diversified isoforms that can form homo- and heterodimers, with each monomer consisting of nine α-helices in an anti-parallel arrangement and they associate through the N-terminal regions to assemble the dimeric protein. Each 14-3-3 dimer is able to interact with two different target proteins at the same time [5–7].

The 14-3-3 proteins in plants function in ubiquitous physiological processes, such as growth and development, cell metabolism, signal transduction, stress responses, postharvest ripening, and nitrogen fixation. In *Arabidopsis*, the T-DNA insertion mutants *14-3-3 μ* and *14-3-3ʋ* result in a delay of flowering under long-day (LD) conditions [8]. The 14-3-3 proteins SGF14c and SGF14l from *Glycine max* play important roles in early developmental stages of soybean nodules [9]. In banana (*Musa acuminata*), *MaGRF*s have significant transcriptional responses during fruit development and postharvest ripening [10]. The 14-3-3 proteins also function in abiotic stress responses [11]. The overexpression of *At14-3-3λ* in cotton, *AtGRF9* in *Arabidopsis*, and *Zea mays GF14–6* in rice (*Oryza sativa*) enhance drought resistance [12–14]. Five *OsGRF*s (*OsGF14b/c/d/e/f*) and three *Vitis vinifera GRF*s (*VviGRF15/17/like2*) show significant expression changes under cold stress in rice and grape, respectively [15, 16].

In recent years, an increasing number of studies on the interactions between 14-3-3 proteins and other proteins have been performed. For example, tomato (*Solanum lycopersicum*) 14-3-3/74 protein interacts with the SELF-PRUNING protein, which is homologous to CENTRORADIALIS in *Antirrhinum* and TERMINAL FLOWER1 in *Arabidopsis*. Furthermore, the overexpression of the *14-3-3/2* and *14-3-3/74* genes complements the loss of function of *SELF-PRUNING* [17, 18]. The overexpression of *GF14c* from *Phyllostachys violascens* in *Arabidopsis* significantly delays flowering time under LD conditions, and PvGF14c interacts with the FLOWERING LOCUS T (FT) homologues PvFT and AtFT in the cytoplasm [19]. The rice FT homologue Hd3a interacts with 14-3-3 proteins to form a complex and binds to the basic leucine zipper transcription factor OsFD1 to form a florigen activation complex (FAC) and induce the transcription of *OsMADS15*, a homologue of *A. thaliana APETALA1* (*AP1*), leading to flowering [8]. This FAC-related phenomenon also exists in *Arabidopsis* [20]. Furthermore, the serine/threonine residue in the C-terminal phosphorylation motif of FD and the arginine residue in the phosphoserine-binding pocket of the 14-3-3 protein are required for the interaction between 14-3-3 and FD proteins [21, 22].

Upland cotton (*Gossypium hirsutum*), an important economic crop, is a major cultivated fiber crop worldwide [23]. The cloning and characterization of the *14-3-3* genes in *G. hirsutum* have attracted attention. The cotton *14-3-3* genes appear to function in fiber cell initiation and elongation, salt and drought stress signaling, and *Verticillium dahlia* resistance [24–28]. At present, the *14-3-3* genes of several plants have been identified, such as *Arabidopsis* [29–31], rice [15, 32], *Medicago truncatula* [33], *Phyllostachys violascens* [19], soybean [34], and foxtail millet (*Setaria italica*) [35]. However, systematic investigations of *14-3-3* family members using genome sequences and of their functions in flowering have not been reported for *G. hirsutum*.

In this study, 82 *GRF* genes were identified from the genome data of *G. herbaceum*, *G. arboreum*, *G. hirsutum*, and *G. raimondii* by genome-wide identification. We analyzed their chromosomal distributions, phylogenic relationships, gene structures, motifs, synteny, duplicated genes, and promoter *cis*-acting elements in detail. The expression profiles of *GhGRF* genes in various organs of ovules and fibers at different developmental stages were comprehensively characterized, and biochemical experiments verified that GhGRF proteins interacted with the cotton FT homologue GhFT and bZIP transcription factor FD homologue GhFD to form a FAC. In addition, our transgenic research showed that *GhGRF3*, *GhGRF6*, *GhGRF9*, *GhGRF14*, and *GhGRF15* were involved in flowering. The present results provide a foundation for the further functional characterization of the *GRF* gene family and the genetic improvement of cotton.

## Results

### Identification and chromosomal distributions of the *GRF* family genes in cotton

A total of 24, 24, 45, and 61 GRF protein sequences were identified from genome databases of four cotton species, *G. herbaceum*, *G. arboreum*, *G. hirsutum*, and *G. raimondii*, respectively, using Hidden Markov Model (HMM) searches. Conserved domain database and Pfam were used to further confirm the presence of the 14-3-3 domain. After removing partial and redundant sequences, 17, 17, 31, and 17 potential *GRF* genes were identified by genome-wide identification from *G. herbaceum*, *G. arboreum*, *G. hirsutum*, and *G. raimondii*, respectively. In total, 31 *G. hirsutum* GRF proteins were named based on their phylogenetic relationships and amino acid sequence similarities with *Arabidopsis* GRFs. If the amino acid sequences from the A or D subgenome had one-to-one similarities with *Arabidopsis* GRF members, they were named the corresponding *Arabidopsis* name, such as AtGRF1 and GhGRF1-A/D. Because there are only 13 GRFs in *Arabidopsis*, the remaining members containing the same 14-3-3 domain were named GhGRF14–17-A/D (Additional file 1: Table S1). GRF proteins recognized from the diploid cotton genomes were named on the basis of the corresponding name in *G. hirsutum* (Additional file 2: Table S2). The GRF proteins identified in cotton ranged from 148 to 421 amino acids. Their molecular weights ranged from 17 to 105 kDa, and the isoelectric points from 4.48 to 6.55 (Additional file 3: Table S3).

Cotton *GRF* genes were not distributed on all 13 chromosomes. In *G. herbaceum*, 17 *GheGRF* genes were located on seven different chromosomes, with chromosome 05 having five genes (*GheGRF1*, *8*, *10*, *11*, and *13*), while chromosomes 01 (*GheGRF2* and *16*), 03 (*GheGRF4* and *6*), 06 (*GheGRF9* and *12*), 07 (*GheGRF7* and *18*), and 13 (*GheGRF14* and *17*) contained two genes each. *GheGRF15* was located on chromosome 04 (Additional file 4: Fig. S1a). In *G. arboreum*, chromosome 10 contained three genes (*GaGRF5*, *8* and *10*), while chromosomes 01 (*GaGRF7* and *13*), 04 (*GaGRF1–1* and *11*), 08 (*GaGRF9* and *14*), 12 (*GaGRF1–2* and *15*), and 13 (*GaGRF12* and *17*) contained two genes each. The genes *GaGRF6*, *4*, and *2* were located on chromosomes 03, 05, and 07, respectively (Additional file 4: Fig. S1b). In *G. hirsutum*, chromosomes A02, D02, A03, A04, D04, D06, and A07 contained one gene each, *GhGRF6-A*, *GhGRF6-D*, *GhGRF4-A*, *GhGRF15-A*, *GhGRF15-D*, *GhGRF9-D*, and *GhGRF7-A*, respectively. Two genes were found on chromosomes A01 (*GhGRF2-A* and *16-A*), D01 (*GhGRF2-D* and *16-D*), A06 (*GhGRF9-A* and *14-A*), A13 (*GhGRF12-A* and *17-A*) and D13 (*GhGRF12-D* and *17-D*). In addition, three genes (*GhGRF3-D*, *5-D*, and *7-D*) were located on chromosome D07, and five genes were on both chromosomes A05 (*GhGRF1-A*, *8-A*, *10-A*, *11-A*, and *13-A*) and D05 (*GhGRF1-D*, *8-D*, *10-D*, *11-D*, and *13-D*) (Additional file 4: Fig. S1c). Similarly, chromosome 09 had the largest number of *GRF* genes (*GrGRF1*, *8*, *10*, *11*, and *13*), followed by chromosome 01 with three genes (*GrGRF3*, *5*, and *7*). Chromosomes 02 (*GrGRF2* and *16*), 05 (*GrGRF6* and *19*), and 13 (*GrGRF12* and *17*) contained two genes each, and the last three genes (*GrGRF18*, *9*, and *15*) were located on the chromosomes 06, 10, and 12, respectively (Additional file 4: Fig. S1d).

### Multiple sequences alignment and phylogenetic analysis of GRF family members in cotton

**Fig. 1:**
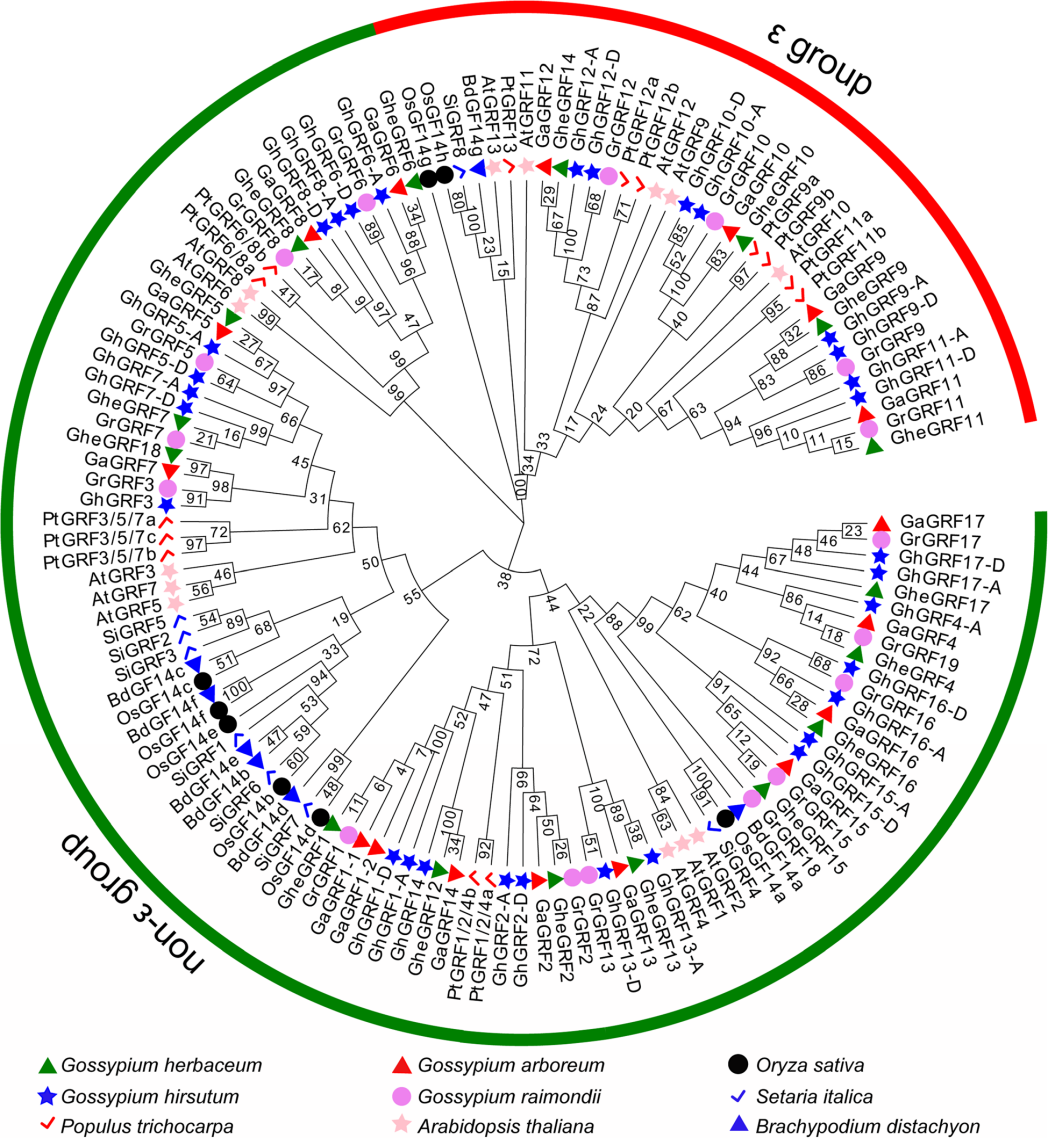
Phylogenetic analysis of the selected plant GRF proteins. Phylogenetic tree was constructed by MEGA5 with neighbor-joining method. Two groups were shown as ε group and non-ε group from alignments of 132 GRF amino acid sequences from *G. herbaceum* (17), *G. arboreum* (17), *G. hirsutum* (31), *G. raimondii* (17), *Arabidopsis* (13), rice (8), *P. trichocarpa* (14), *B. distachyon* (7), and foxtail millet (8)

The multiple amino acid sequence alignment revealed nine α-helices in cotton GRF protein secondary structures, of which α3 and α4 were the longest (Additional file 5: Fig. S2). To examine the evolutionary relationships among *GRF* gene family members in cotton, we constructed a phylogenetic tree with the neighbor-joining [36] method using 17 *G. herbaceum*, 17 *G. arboreum*, 31 *G. hirsutum*, 17 *G. raimondii*, 13 *Arabidopsis*, 8 rice, 14 *Populus trichocarpa*, 7 *Brachypodium distachyon*, and 8 foxtail millet members’ GRF amino acid sequences (Fig. 1). The 132 GRF proteins were classified into two major groups: ε and non-ε. According to the phylogenetic relationship, four GRF proteins of *G. herbaceum* (GheGRF9, 10, 11, and 14), *G. arboreum* (GaGRF9, 10, 11, and 12) and *G. raimondii* (GrGRF9, 10, 11, and 12), along with eight GRF proteins from *G. hirsutum* (GhGRF9-A, 10-A, 11-A, 12-A, 9-D, 10-D, 11-D, and 12-D), five from *Arabidopsis* (AtGF9, 10, 11, 12, and 13), two from rice (OsGF14g and h), seven from *P. trichocarpa* (PtGRF9a, 9b, 11a, 11b, 12a, 12b, and 13), one form *B. distachyon* (BdGF14g), and one from foxtail millet (SiGRF8) were encoded by genes belonging to the ε group. The other 96 *GRF* genes belonged to the non-ε group.

### Analyses of cotton *GRF* gene structures and GRF protein motifs

**Fig. 2:**
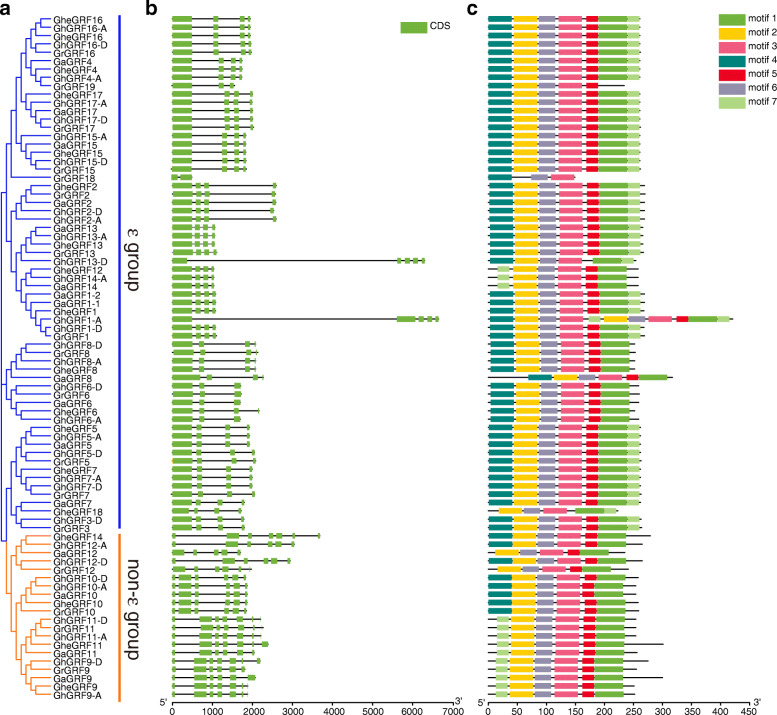
Phylogenetic relationships, gene structure and conserved protein motif analyses of *GRF* genes from cotton. **a** A neighbor-joining phylogenetic tree of 82 GRF proteins in cotton was constructed using MEGA5.1 software and the bootstrap consensus tree was inferred from 1000 replicates. **b** Exon–intron structure of cotton *GRF* genes. **c** Distributions of seven conserved motifs in cotton *GRF* genes. The sequence information for each motif is provided in Additional file 6: Table S4

Gene structural divergence plays a main role in the evolution of gene families. To characterize the gene structures of the *GRF* genes identified from four *Gossypium* species, a phylogenetic tree was constructed (Fig. 2a), and the exon–intron structures of 82 *GRF* genes were analyzed. The *GRF* genes contained two to seven exons, with 20 genes in the non-ε group possessing five to seven exons, while the ε group members possessed two to five exons (Fig. 2b). The diversity of the exon–intron structures between the ε and non-ε groups in cotton indicated differences in the expansion and evolution of the ε and non-ε groups of cotton *GRF* genes. In the ε group, genes composed of five exons were from the allotetraploid *G. hirsutum*, such as *GhGRF1-A* and *GhGRF13-D*, suggesting that some ε group members obtained additional exons during the polyploidization process.

Seven conserved motifs were identified from cotton GRF proteins using an online MEME program [37] and further annotated by InterPro [38]. The lengths of motifs ranged from 21 to 50 amino acids, and motifs 1, 2, 3, and 4 were annotated as 14-3-3 protein domains (Additional file 6: Table S4). Motifs 3 and 6 existed in all of the GRF proteins of cotton, showing high conservation, indicating that they may be requisite for gene function. In the ε group, most GRF proteins contained motifs 4 and 7 at the N and C termini, respectively. Most of the non-ε group had either motif 4 or 7 at the N terminus, and they all had motif 1 at the C terminus (Fig. 2c). The differences in motif structures between ε and non-ε groups may reflect their functional diversity.

### Synteny and duplicated gene analysis of cotton *GRF* genes

**Fig. 3:**
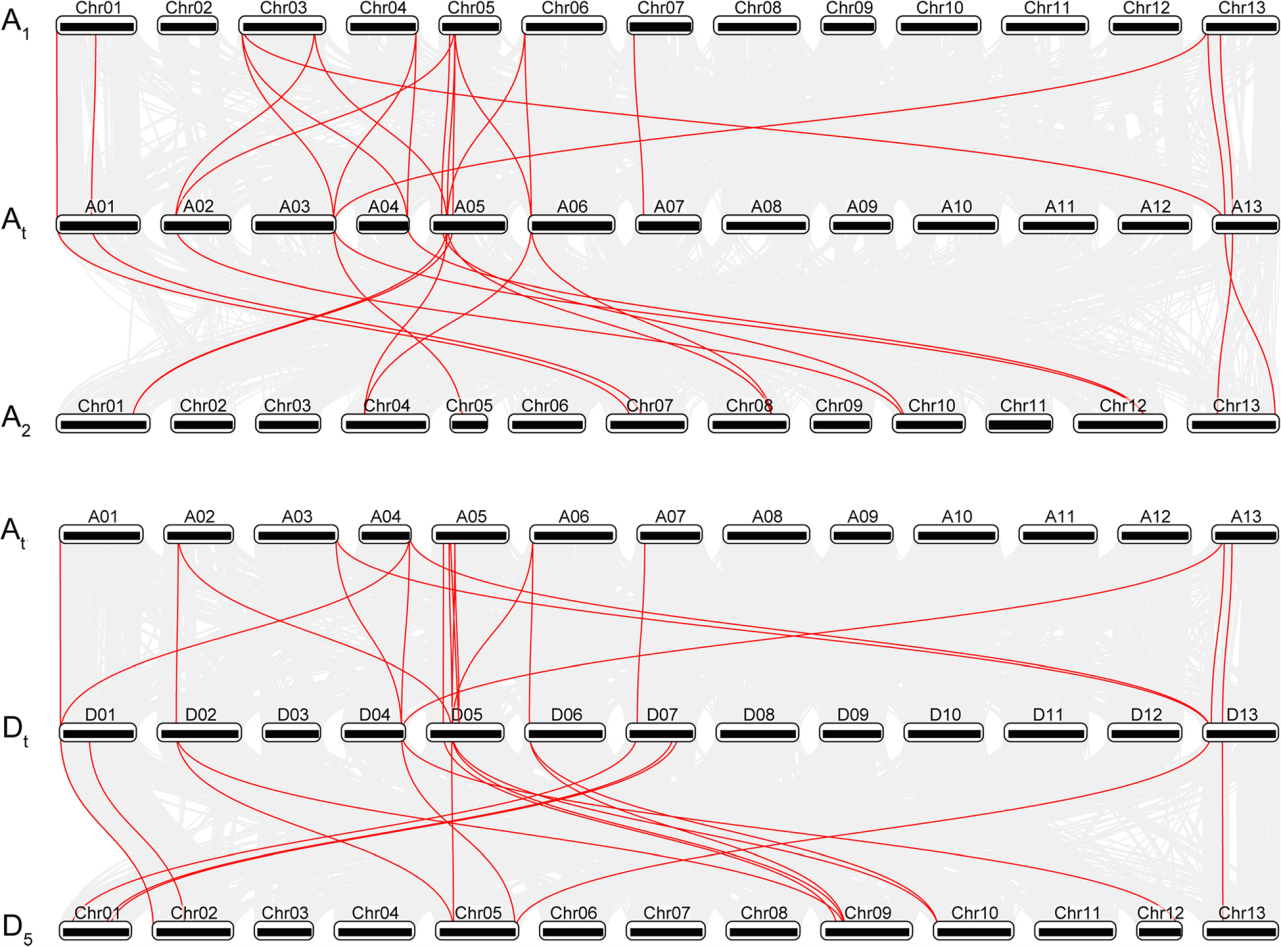
Synteny analysis of *GRF* genes between *G. hirsutum* and the other three *Gossypium* spp. species. Gray lines in the background indicate the collinear blocks within *G. hirsutum* and other three cotton genomes, while the red lines highlight the syntenic *GRF* gene pairs. The specie names with the prefixes ‘A_1_’, ‘A_t_’, ‘A_2_’, ‘D_t_’, and ‘D_5_’ represent *G. herbaceum*, A subgenomes of *G. hirsutum*, *G. arboreum*, D subgenomes of *G. hirsutum* and *G. raimondii*, respectively

To deduce the phylogenetic mechanisms of the *GRF* family in *G. hirsutum*, we constructed comparative syntenic maps of *G. hirsutum* with the other three *Gossypium* species, including the A subgenome of *G. hirsutum* with the diploid A-genome cotton species *G. herbaceum* and *G. arboreum*, and the D subgenome of *G. hirsutum* with the A subgenome and diploid D-genome *G. raimondii* (Fig. 3). A total of 20 and 15 *GRF* genes from A_t_-subgenome of *G. hirsutum* exhibited syntenic relationships with those in *G. herbaceum* and *G. arboreum*, respectively, while 19 and 17 *GRF* genes from the D_t_-subgenome of *G. hirsutum* showed syntenic relationships with those in the A subgenome and *G. raimondii*, respectively (Fig. 3 and Additional file 7: Table S5). Some *GRF* genes formed two or three syntenic gene pairs (particularly between the A_t_-subgenome of *G. hirsutum* and the D subgenome of *G. hirsutum*, and the A_t_-subgenome of *G. hirsutum* and *G. herbaceum*), such as *GhGRF4-A* and *GhGRF15-A* from the A_t_-subgenome of *G. hirsutum*. We hypothesize that these genes played crucial roles in the evolution of the *GRF* gene family.

Gene duplication is generally considered to be a necessary source of material for the generation of evolutionary novelties and the production of new gene functions [39, 40]. A genome duplication analysis revealed that 12, 12, 49, and 15 paralogous pairs were duplicated *GRF* gene pairs (Additional file 8: Table S6). To better understand the selective evolutionary pressure on this gene family, the non-synonymous (*Ka*) and synonymous (*Ks*) substitution ratios of these gene pairs were calculated (Additional file 7: Table S5 and Additional file 8: Table S6). The paralogous pairs in all four cotton species had *Ka*/*Ks* < 1, suggesting that the cotton *GRF* gene family has undergone strong purifying selection during evolution (Additional file 9: Fig. S3a). The syntenic gene pairs, except for the pair *GhGRF9-D* and *GrGRF9*, which were from D subgenome of *G. hirsutum* and *G. herbaceum*, respectively, experienced positive selection (*Ka*/*Ks* > 1), having evolved under purifying selection (*Ka*/*Ks* < 1) (Additional file 9: Fig. S3b).

### Prediction of *cis*-acting elements in cotton *GRF* promoters

To determine whether the regulatory mechanism was influenced by *cis*-acting elements in *GRF* promoters, the sequences 2.0-kb upstream of the translation start sites were analyzed. A number of putative *cis*-elements in the four *Gossypium* species may be involved in various responses, such as to light, anaerobic induction, methyl jasmonate (MeJA), abscisic acid, salicylic acid, and auxin. Among these elements, light responsive element was the most abundant responses, followed by anaerobic induction and MeJA-responsive (Additional file 10: Fig. S4).

A total of 25 *cis*-acting elements related to light responsiveness were recognized, including an AE-box, GTGGC-motif, GT1-motif, Box 4, TCT-motif, G-Box, Sp1, MRE, and I-box. Some gibberellin-responsive elements were found, including a TATC-box, P-box, and GARE-motif. Two MeJA-responsive elements (CGTCA-motif and TGACG-motif), auxin-responsive elements (AuxRR-core and TGA-element), endosperm expression elements (GCN4-motif and AAAC-motif), and salicylic acid-responsive elements (TCA-element and SARE) were identified. Anaerobic induction elements, such as AREs were observed. In addition, other abiotic stress-response factors, such as an MBS, circadian, LTR, WUN-motif, ARE, and TC-rich repeats, were also present (Additional file 11: Fig. S5).

### Expression profiles of *GRF* genes in *G. hirsutum*

**Fig. 4:**
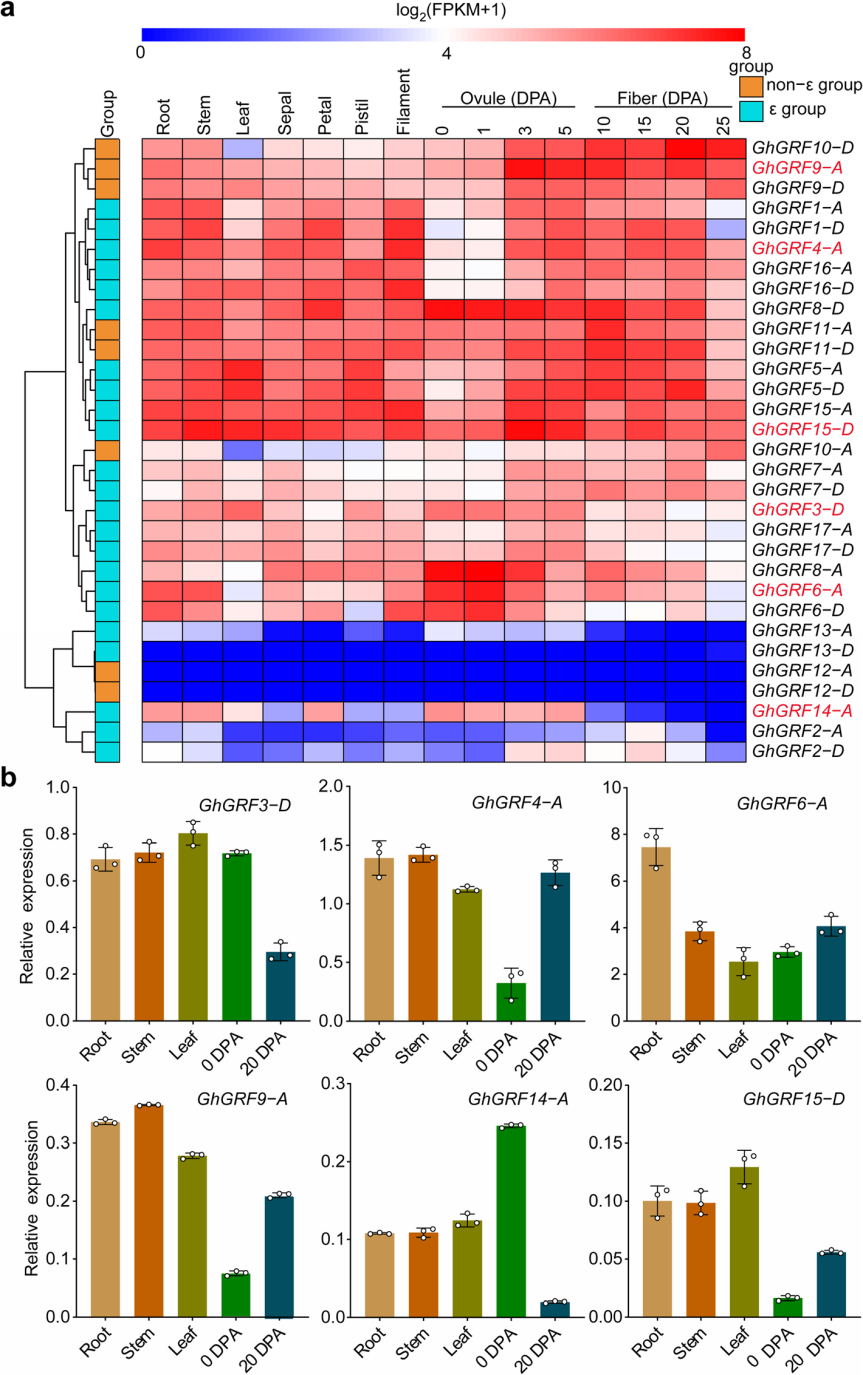
Expression profiles of *GhGRF* genes in 15 tissues. **a** Roots, stems, leaves, sepals, petals, pistils, filaments, fiber-bearing ovules on 0, 1, 3, and 5 d post-anthesis (DPA), and fibers on 10, 15, 20, and 25 DPA were sampled. **b** Expression analysis of six *GhGRF* genes marked with red fonts in **a** by qRT-PCR in five samples

To analyze the expression patterns of the identified *GRF* genes in cotton, 15 tissues, including roots, stems, leaves, sepals, petals, pistils, filaments, ovules, and fibers, at different developmental stages were selected and their expression levels analyzed using published RNA-seq data (PRJNA490626) [41]. In total, 31 *GhGRF*s exhibited differential expression levels in the investigated tissues (Fig. 4a). The values of fragments per kilobase of exon model per million mapped reads (FPKM) for four *GhGRF* genes (*GhGRF12*-*A*, *GhGRF12*-*D*, *GhGRF13*-*D*, and *GhGRF14*-*A*) were equal to 0 in some tissues and two genes (*GhGRF2*-*A* and *GhGRF13*-*A*) had 0 < FPKM < 1. The FPKM values of 25 *GhGRF*s detected in all 15 samples were greater than 1 (Additional file 12: Table S7). Fifteen *GhGRF* genes were highly expressed in all the tested tissues, including *GhGRF5*-*A*, *GhGRF8*-*D*, *GhGRF11*-*A*, *GhGRF11*-*D*, *GhGRF15*-*A*, and *GhGRF15*-*D*, which suggested that they are involved in the whole process of cotton growth and development. Nine *GhGRF* genes were also expressed in all the tissues at relative lower expression levels compared with the 15 highly expressed genes. However, the expression levels of *GhGRF*8-*A* and *GhGRF6*-*A*, and *GhGRF6*-*D* were similar to those of the 15 genes in ovules at 0 and 1 d post-anthesis (DPA). Seven *GhGRF* genes showed lower expression levels than these genes, but *GhGRF14-A* was highly expressed in roots, stems, petals, and ovules. Thus, they might play important roles as tissue-specific regulators.

Subsequently, quantitative real-time PCR (qRT-PCR) experiments were performed for six representative *GhGRF* genes to further verify the reliability of the transcriptome data. Roots, stems, leaves, ovules at 0 DPA, and fibers at 20 DPA were used as samples (Fig. 4b). *GhGRF3-D* and *GhGRF14-A* were highly expressed in roots, stems, leaves, and 0-DPA ovules, while *GhGRF4-A*, *GhGRF9-A*, and *GhGRF15-D* were highly expressed in roots, stems, leaves, and 20-DPA ovules. *GhGRF6-A* showed the lowest expression level in leaves. The qRT-PCR results were mostly consistent with the transcriptome data, and the ubiquitous expression profiles suggested that *GRF* genes are involved in diverse developmental controls in cotton.

### Interactions of GhFT and GhFD with each GhGRF protein

**Fig. 5:**
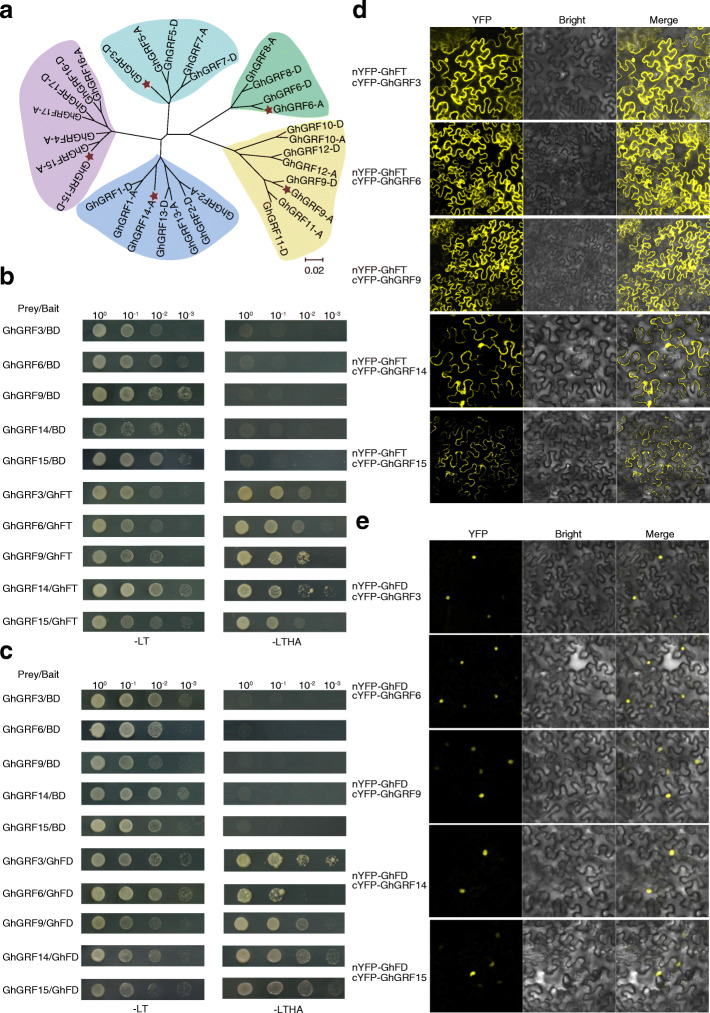
Interactions of GhFT and GhFD with each GhGRF protein. **a** Phylogenetic analysis of 31 GhGRF proteins. Red pentagram represent the selected GhGRF proteins used to validate interaction with GhFT. **b** Y2H experiment between GhFT and each GhGRF protein. **c** Y2H experiment between GhFD and each GhGRF protein. Plasmids transformed into AH109 yeast strains were screened on the -LT and -LTHA medium. -LT and -LTHA medium represent SD/−Trp-Leu and SD/−Trp-Leu-His-Ade medium. **d** BiFC verification of interactions between GhFT and each GhGRF protein. **e** BiFC verification of interactions between GhFD and each GhGRF protein. Constructs of *cYFP-GhGRF*s and *nYFP-GhFD* were co-transformed into *N. benthamiana* leaf epidermal cells

The formation of a FAC containing Hd3a, FD, and 14-3-3 is critical in mediating flowering [7, 20, 22]. A phylogenetic tree analysis showed that 31 GhGRF homologues clearly divided into five branches (Fig. 5a). To determine whether these GhGRFs form FACs in upland cotton, we selected a representative GhGRF protein from each of the five different branches and performed yeast two-hybrid (Y2H) experiments. GhFT and GhFD in upland cotton interacted with the selected GhGRF proteins, GhGRF3-D, GhGRF6-A, GhGRF9-A, GhGRF14-A, and GhGRF15-D, in vitro (Fig. 5b and c). Subsequently, we further verified their interactions using bimolecular fluorescence complementation (BiFC) experiments in the epidermal leaves of *Nicotiana benthamiana* (Fig. 5d and e). Moreover, the GhFT–GhGRF interaction was detected in the cytoplasm and nucleus, and the fluorescence signal of GhFD–GhGRF proteins was localized in the nucleus. Because GhGRF interacted with GhFT and GhFD, we, therefore, inferred that these five GhGRF proteins interact with GhFT and GhFD to form five different FACs. In addition, other GhGRF proteins also interacted with GhFT as assessed by Y2H experiments (Additional file 13: Fig. S6), suggesting that GhGRFs form FACs with GhFT and GhFD.

### The effects of silencing five *GhGRF* genes on flowering time in *G. hirsutum*

**Fig. 6:**
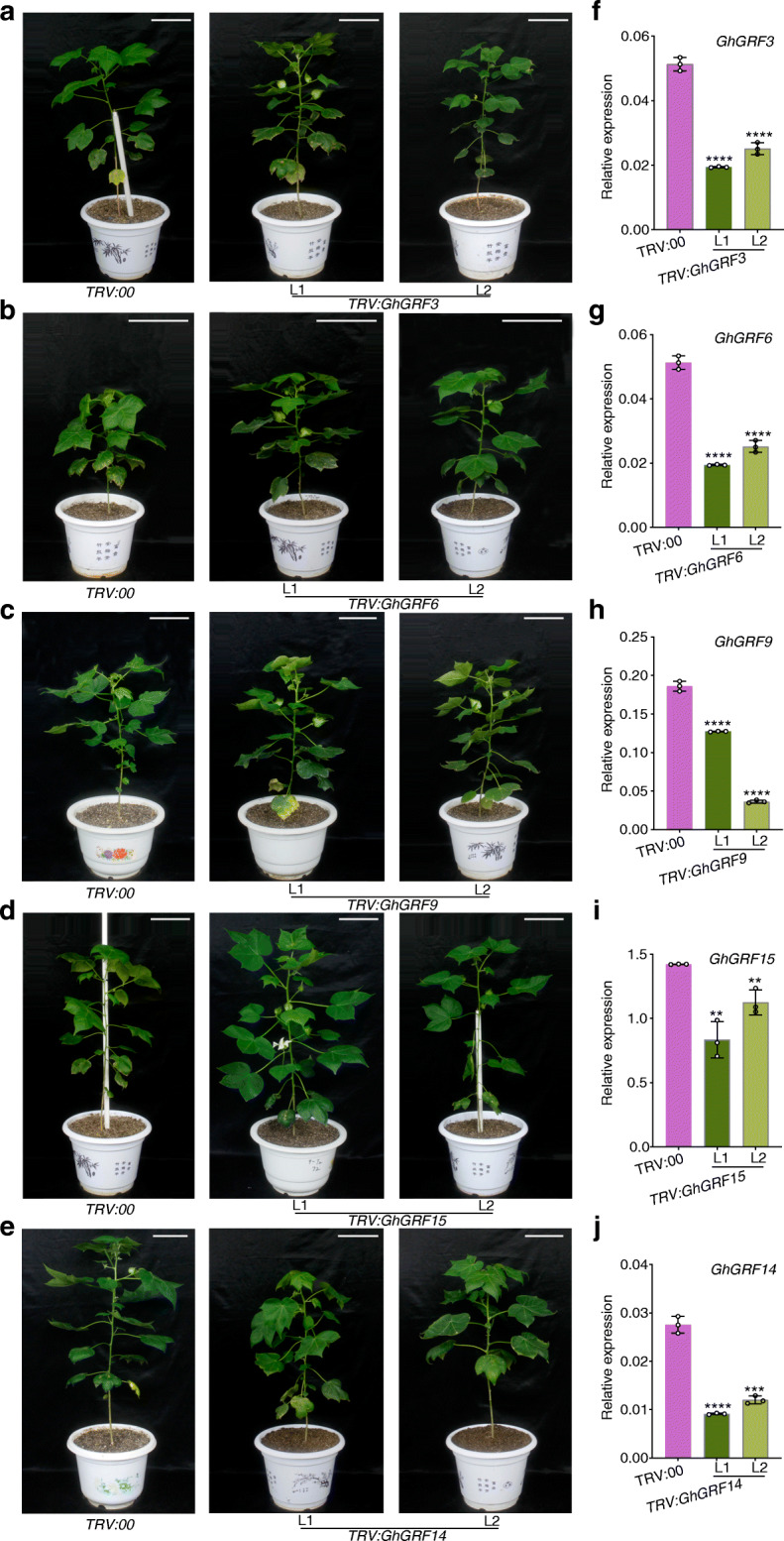
Phenotypical analyses of silencing of five *GhGRF* genes in *G. hirsutum*. Phenotypes of *TRV:00* and *TRV:GhGRF3* (**a**), *TRV:GhGRF6* (**b**), *TRV:GhGRF9* (**c**), *TRV:GhGRF15* (**d**), and *TRV:GhGRF14* (**e**). Relative expression of *GhGRF3* (**f**), *GhGRF6* (**g**), *GhGRF9* (**h**), *GhGRF15* (**i**), and *GhGRF14* (**j**) in *TRV:00* and *TRV:GhGRF*s plants. *Ubiquitin7* (*GhUBQ7*, GenBank accession no. DQ116441) gene was used as an internal reference transcript. Values are means ± *SD* (*n* = 3). Asterisks indicate significant differences between control *TRV:00* and *TRV:GhGRF*s silencing lines (Student’s *t* test, **P < 0.01, ***P < 0.001, ****P < 0.0001). Scale bar, 30 cm

To explore the possible roles of *GhGRF* genes in cotton, we performed a virus-induced gene silencing (VIGS) assay. The flowering times in *GhGRF3*, − *6*, − *9*, and − *15*-silenced plants were earlier than that of control plants (Fig. 6a–d and Additional file 14: Fig. S7a–d). However, the flowering time in *GhGRF14*-silenced plants was much later than that of control plants (Fig. 6e and Additional file 14: Fig. S7e). Gene transcription analyses using qRT-PCR revealed that the expression level of each *GhGRF* significantly decreased in the corresponding silenced plants (Fig. 6f–j). Moreover, the expression levels of floral meristem-identity genes, *AP1* homologues *GhAP1* (Gh_D13G0878) and *SUPPRESSOR OF OVEREXPRESSION OF CONSTANS 1* (*SOC1*) homologues *GhSOC1* (Gh_A11G0755) in cotton, were upregulated in *GhGRF3*, − *6*, − *9*, and − *15*-silenced plants, whereas they were downregulated in the *GhGRF14*-silenced plants (Additional file 14: Fig. S7f–o).

### The effects of the ectopic expression of five *GhGRF* genes on flowering time in transgenic *Arabidopsis*

**Fig. 7:**
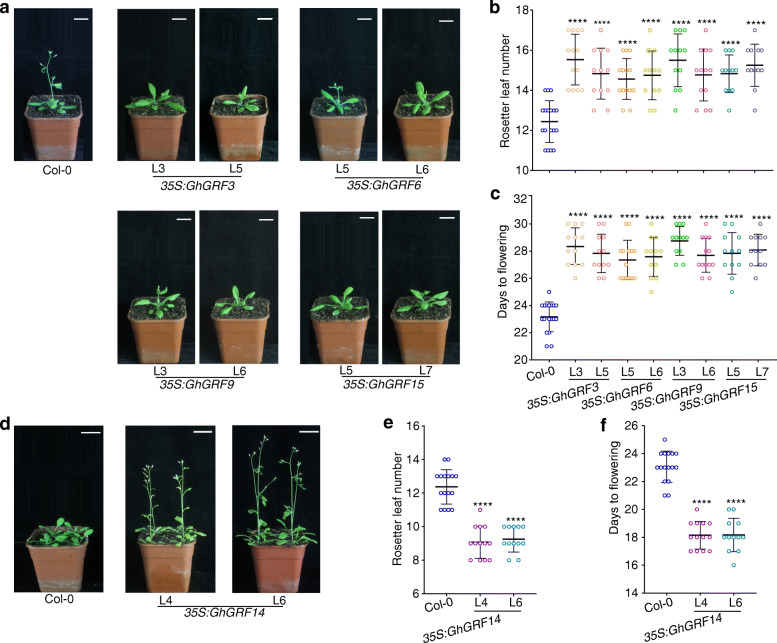
Phenotypical analyses of transgenic *Arabidopsis* plants that ectopically expressed five *GhGRF* genes under Long-day conditions. **a** Phenotype of 23 d wild-type *Arabidopsis* (Col-0) and transgenic plants with *35S:GhGRF3*, *35S:GhGRF6*, *35S:GhGRF9*, and *35S:GhGRF15*. **b** The number of rosette leaves in Col-0 and four homologous transgenic plants at anthesis stage. **c** The days to flowering in Col-0 and four homologous transgenic plants at anthesis stage. **d** Phenotype of 18 d Col-0 and transgenic plants overexpressing with *35S:GhGRF14*. **e** The number of rosette leaves in Col-0 and homologous *35S:GhGRF14* transgenic plants at anthesis stage. **f** The days to flowering in Col-0 and homologous *35S:GhGRF14* transgenic plants at anthesis stage. At least 12 plants for each line were scored (Student’s *t* test, ****P < 0.0001). Scale bar, 2 cm

To further demonstrate the functions of the five *GhGRF* genes, we constructed overexpression vectors driven by the Cauliflower mosaic virus *35S* promoter and generated corresponding transgenic *Arabidopsis* plants. The transgenic plants overexpressing *GhGRF3*, − *6*, − *9*, and − *15* experienced delayed flowering compared with wild-type (Col-0) under LD conditions (Fig. 7a). The numbers of rosette leaves and flowering times in *GhGRF3*, − *6*, − *9*, and − *15* transgenic plants were greater than those of wild-type (Fig. 7b and c). However, *GhGRF14*-overexpressing plants exhibited early flowering compared with wild-type (Col-0) (Fig. 7d), and the numbers of rosette leaves and flowering time were less than in wild-type (Fig. 6e and f). To investigate whether the flowering time was related to the expression levels of *GhGRF* genes in transgenic *Arabidopsis*, the transcript levels of *GhGRF* genes were detected in the homologous transgenic lines by qRT-PCR. The expression levels of the *GhGRF* genes were significantly increased in the transgenic plants (Additional file 15: Fig. S8a and b). The flower meristem-identity genes, *AtAP1* and *AtSOC1*, were downregulated in *35S:GhGRF3*, *35S:GhGRF6*, *35S:GhGRF9*, and *35S:GhGRF15* transgenic plants, while they were upregulated in *GhGRF14* overexpressing plants (Additional file 15: Fig. S8c–f), which correlated with their flowering times. These results were consistent with the effect of silencing the five *GhGRF* genes in the regulation of flowering, suggesting that they have roles in flowering regulation.

## Discussion

The 14-3-3 proteins are highly conserved in eukaryotes [1], and can form homo- and heterodimers [42], which are able to interact with two different target proteins at the same time to form complexes [5–7]. Therefore, they have great effects on many plant biological progresses. In recent years, *14-3-3* families have been identified and studied in many plant species [16, 34, 35]. To date, although research on *14-3-3* family members in *G. hirsutum* has been performed, it mainly focused on the roles of *14-3-3* in fiber development and stress responses [24–28]. However, comprehensive systematic analyses and investigations into their functions in flowering are still limited.

### The conserved and diverse functions of the *GRF* gene family in cotton

In this study, genome-wide analyses identified 17, 17, 31, and 17 *GRF* genes from *G. herbaceum*, *G. arboreum*, *G. hirsutum*, and *G. raimondii*, respectively, in the CottonGen database. The discovery implied that *GRF* genes had experienced expansion in tetraploid *G. hirsutum* compared with that in diploid *G. herbaceum*, *G. arboreum*, and *G. raimondii*. On the basis of phylogenetic relationships with other plants, the GRF proteins were classified into ε and non-ε group (Fig. 1), which was consistent with previous studies in rice [32], *Arabidopsis* [31], populus [43], banana [10], and soybean [34]. A gene structural analysis revealed that non-ε group members commonly possessed less exons and introns than ε group members (Fig. 2b), which was also found in other species [31, 32, 44], and a motif analysis indicated that ε and non-ε groups had different motif structures, which suggested their functional diversity (Fig. 2c).

Some *GRF* genes formed more than one syntenic gene pair, such as *GhGRF4-A* and *GhGRF15-A*, which implied they played vital roles in evolution (Fig. 3 and Additional file 7: Table S5). The number of duplicated gene pairs in upland cotton was more than that in diploid cotton. This was related to the expansion of *GRF* genes in *G. hirsutum,* which had all undergone purifying selection (Additional file 8: Table S6 and Additional file 9: Fig. S3a). Putative *cis*-elements of *GRF* genes in four *Gossypium* species participated in various responses, suggesting their functional diversity (Additional file 10: Fig. S4).

Expression profiles revealed that *GhGRF*s were differentially expressed in all of the investigated tissues (Fig. 4a). In total, 15 *GhGRF* genes were highly expressed in all the tested tissues, followed by nine *GhGRF* genes, suggesting that these genes participated in the whole process of cotton growth and development. Seven *GhGRF* genes were expressed lower or not expressed in some tissues. Specially, *GhGRF14-A* was highly expressed in roots, stems, petals, and ovules. In addition, some *GhGRF*s exhibited tissue-specific expression, such as *GhGRF6*-*A*/*D*, *GhGRF8*-*A*, and *GhGRF14-A*. This phenomenon also existed in some *14-3-3* genes of other plants. The expression of *General regulatory factor1-G-box factor 14-3-3 homolog isoform chi* (*GRF1-GF14x*) from *Arabidopsis* has been detected in roots, flowers, siliques, and imbibed seeds, but not in leaves or cotyledons [3]. In switchgrass (*Panicum virgatum*), *PvGF14a* is expressed highly in lignified organs, while *PvGF14r* is highly expressed in the inflorescence meristem and florets of inflorescence [45]. In grape, *VviGRF12* shows high expression levels in some floral organs [16]. The diversity of the *GhGRF* expression levels suggested that functional differentiation may have emerged during evolution.

### The diversified ternary complexes formed by GhFT, GhFD, and each GRF protein in cotton

The 14-3-3 proteins bind to flowering time regulatory proteins, such as FT [46–48] and *CONSTANS* [8]. GhFT localizes to the cytoplasm and nucleus, and FT interacts with FD in the nucleus [49, 50]. In this study, we determined that GhGRF3/6/9/14/15 interacted with GhFT in the cytoplasm and nucleus (Fig. 5d), whereas they interacted with GhFD in the nucleus (Fig. 5e). The results indicated that GhFT, GhFD, and individual GhGRF proteins form different FACs in the nucleus. The formation of FACs has been reported in other species. In rice, a 14-3-3 protein interacts with Hd3a in the cytoplasm and is translocated to the nucleus where it binds OsFD to form a FAC. If OsFD1 is recruited, then the function of FT–14-3-3–FD1 is to promote flowering, while if OsFD2 is recruited, then the FAC plays a role in rice leaf development [22]. In *Arabidopsis*, the FT protein binds to the 14-3-3 and FD proteins to form an active FT–14-3-3–FD complex that promotes flowering [20]. In potato (*Solanum tuberosum*), StSP6A, an Hd3a homolog, and StFDL1, a potato FD-like protein, interact with St14-3-3 s in stolon tips to form FAC-like complexes that initiate tuber formation [51]. The multifunctionality of FACs provides a direction to further study the functional diversity of *14-3-3* genes. Moreover, Y2H experiments confirmed that all the identified GhGRF proteins interacted with GhFT (Additional file 13: Fig. S6), suggesting that GhGRF proteins interact with GhFT and GhFD to form FACs that exercise diverse functions. This hypothesis needs to be further studied.

### FACs formed through GRFs either promote or delay flowering in cotton

The FAC composed of florigen FT, 14-3-3 protein, and FD promotes flowering by activating downstream target genes [7, 20]. Here, we showed that upland cotton GhGRF3/6/9/14/15 formed five FACs with GhFT and GhFD in the nucleus and that the *GhGRF3/6/9/15* silencing in cotton promoted the expressions of *GhAP1* and *GhSOC1*, resulting in early flowering. In contrast, *GhGRF14* silencing suppressed the expressions of *GhAP1* and *GhSOC1*, resulting in late flowering (Fig. 6 and Additional file 14: Fig. S7). Moreover, the overexpression of *GhGRF3/6/9/15* inhibited the expression of *AtAP1* and *AtSOC1*, causing late flowering, while *GhGRF14* activated *AtAP1* and *AtSOC1* and promoted early flowering under LD conditions (Fig. 7 and Additional file 15: Fig. S8). These data suggested that GhGRFs, as components of the FACs, were essential for controlling flowering time in cotton.

**Fig. 8:**
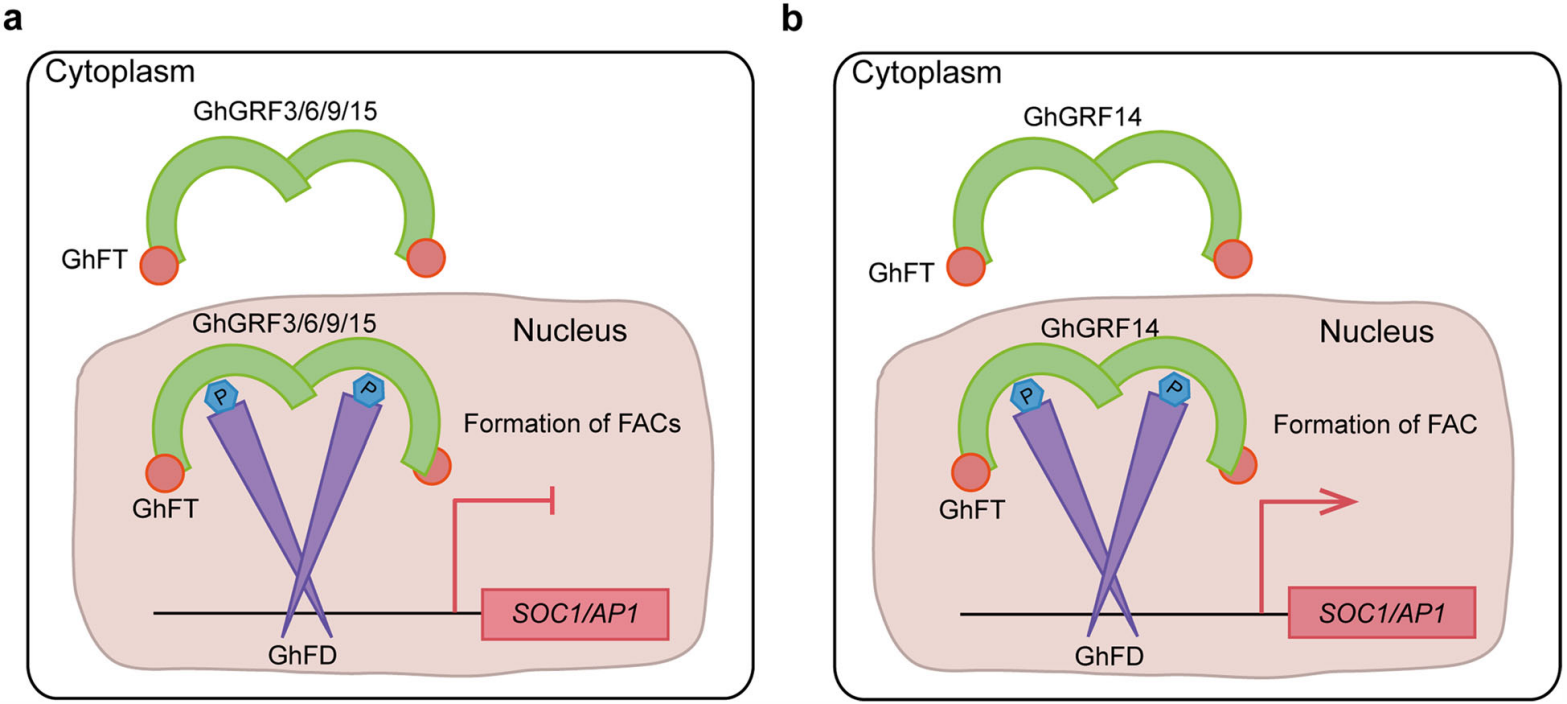
Schematic diagram of the FACs functioning in flowering regulation in cotton. **a** The FACs formed by GhFT, GhFD, and each GhGRF protein, such as GhGRF3, 6, 9, and 15, inhibit the expression of *AP1* and *SOC1* homologues, causing late flowering. **b** The FAC formed by GhFT, GhFD, and GhGRF14 promotes the expression of *AP1* and *SOC1* homologues, resulting in early flowering

Our model of the ternary FACs composed of GhFT, GhFD, and each of the five GhGRF proteins implies that the roles of the FACs in regulating flowering were determined by the GhGRF protein present (Fig. 8). When the interaction occurred between GhFT, GhFD, and GhGRF3/6/9/15, FACs inhibited flowering by downregulating the expression of flower identity genes, such as *AP1* and *SOC1*. However, when the FAC was formed by the interaction between GhFT, GhFD, and GhGRF14, the transcription of the *AP1* and *SOC1* homologues were upregulated to promote flowering. Our findings suggested that GhGRFs regulate flowering time by forming FACs with GhFT and GhFD. In summary, we determined that FACs in cotton function as either activators or repressors of flowering, and we revealed a mechanism of flowering regulation in *G. hirsutum*.

## Conclusions

In this study, 82 *GRF* genes were identified from four cottons (*Gossypium* spp.) by genome-wide analyses. All the GRF proteins contained the conserved 14-3-3 domain and were classified into two groups on the basis of their phylogenetic relationships with GRFs in other plant species. Their structural characteristics, evolution, *cis*-acting elements, and expression profiles revealed the conservation and diversity of *GRF* gene functions in cotton. Furthermore, we showed that GhGRFs interact with GhFT and GhFD proteins to form FACs in the nucleus, which function either in promoting or inhibiting flowering by regulating the expression of floral meristem-identity genes, such as *AP1* and *SOC1* homologues. The results indicate the functional diversification of FACs in cotton and lay a solid foundation for further studies on the functional mechanisms of *GhGRF*s.

## Methods

### Plant materials

Cotton seeds (*G. hirsutum* L. cv. XLZ 33) were field-grown under natural conditions during the summer of 2019 in Shihezi (Xinjiang, China). The seed sterilization and cultivation methods, as well as the growth conditions for *A. thaliana* Columbia (Col-0) and transgenic plants used in this study, were as previously described [49, 52]. Collection of plant or seed specimens complied with relevant institutional, national, and international guidelines and legislation.

For gene expression analyses, the fresh leaves of Col-0 and the transgenic lines were harvested under LD conditions in a plant growth chamber. All collected samples were frozen immediately in liquid nitrogen and stored at − 80 °C.

### RNA extraction and qRT-PCR analyses

Total RNA for each sample was isolated using the RNAprep pure Plant Kit (Tiangen, Beijing, China) and treated with RNase-free DNase (Tiangen) in accordance with the manufacturer’s protocol. The quality, quantity, and integrity of the total RNA extracted were assessed as previously described [49]. Total RNA was reversed transcribed into cDNA using the Superscript First-Strand Synthesis System (Invitrogen, Carlsbad, CA, USA). The qRT-PCR using the SYBR Green Master Mixture (CWBIO, Beijing, China) on an Applied Biosystems 7500 Fast Real-Time PCR System (Life Technologies, Foster City, CA, USA) were performed as previously described [53]. The PCR amplification system and program were as described previously [52]. Primers used in this study are listed in Additional file 16: Table S8. All qRT-PCR assays were repeated three times as independent biological replicates. Relative gene expression levels were calculated using the 2^–∆Ct^ method [54].

### Identification of the *GRF* gene family in cotton

The A_1_ genome of diploid *G. herbaceum* [55], the A_2_ genome of diploid *G. arboreum* [56], the (AD)_1_ tetraploid genome of *G. hirsutum* [57] and the D_5_ genome of diploid *G. raimondii* [58] were downloaded from Cottongen (https://www.cottongen.org/). The HMM document, downloaded from Pfam database (http://pfam.sanger.ac.uk/), was used to identify the *GRF* genes in genome database of four cotton species using Simple HMM Search in TBtools [59]. All predicted GRF protein sequences were submitted to NCBI Batch CD-Search (http://www.ncbi.nlm.nih.gov/Structure/cdd/wrpsb.cgi) [60] and Pfam (https://pfam.xfam.org) [61] to confirm the 14-3-3 domain. Partial and redundant sequences were all manually removed. MapInspect software (http://mapinspect.software.informer.com/) was used to locate those genes on chromosomes. Furthermore, the molecular weight and isoelectric point of GRF proteins were determined using ExPASy (http://web.expasy.org/compute_pi/) [62].

### Multiple sequence alignment and phylogenetic analysis

The amino acid sequences of the GRF family members identified in cotton were aligned using the CLUSTALX program [63]. The full-length amino acid sequences of 14-3-3 s derived from *Arabidopsis*, rice, *P. trichocarpa*, *B. distachyon*, foxtail millet, and cotton were used for phylogenetic analysis using MEGA5.1 [36] by the Neighbor–Joining method with 1000 bootstrap replications. Finally, the online tool Evolview (http://www.evolgenius.info/evolview/) [64], was used to clarify and construct the phylogenetic tree.

### Gene structure, conserved motif, and promoter analyses

The exon–intron structures of the *GRF* genes were generated using the Gene Structure Display Server version 2.0 (http://gsds.cbi.pku.edu.cn/) [65]. MEME software (http://meme-suite.org/tools/meme) [37] was used to identify conserved cotton GRF protein motifs. It also determined that the maximum number of motifs was seven and optimum width was from 6 to 50. The predicted motifs in cotton GRF proteins were further annotated using the InterPro database (http://www.ebi.ac.uk/interpro/) [38]. The PlantCare database (http://bioinformatics.psb.ugent.be/webtools/plantcare/html/) [66] was used to search potential *cis*-regulatory elements in the 2.0-kb promoter region.

### Syntenic and gene duplication analyses

To display the syntenic relationship of *GRF* genes between *G. hirsutum* and the other three analyzed cottons species, syntenic maps were constructed using the Multiple Synteny Plot software from TBtools [59]. BLASTp search with E-value < 1 × 10^− 5^ were performed to identify duplicated *GRF* genes. To be defined as duplicated genes, the following two criteria had to be met: the aligned region of two sequences covered > 80% of the longer sequence and the identity of the aligned regions > 90% [67]. The *Ka* and *Ks* substitution rates of orthologous and homologous pairs of *GRF* genes were calculated using DnaSP software [68]. The selective pressure was analyzed using *Ka*/*Ks* ratio.

### Expression analyses of the *GhGRF* genes using RNA-Seq

To obtain the expression profiles of *GhGRF* genes, RNA-seq data of *G. hirsutum* TM-1 were downloaded from NCBI (https://www.ncbi.nlm.nih.gov/bioproject/PRJNA490626) [41]. The expression levels of *GhGRF* genes in 15 tissues were represented by log-transformed FPKM (log_2_[FPKM+ 1]) values. The R package “pheatmap” was used to generate the expression patterns of the *GhGRF* genes in these tissues.

### Plasmid construction and genetic transformation of *Arabidopsis*

The coding sequences of the five *GhGRF* genes were independently cloned into the pMD19-T vector, and positive plasmids containing each gene, as identified by sequences analyses, were then introduced into the plant binary vector *pCAMBIA2300-35S-OCS* [49] to generate *35S:GhGRF3*, *35S:GhGRF6*, *35S:GhGRF9*, *35S:GhGRF14*, and *35S:GhGRF15*. All of the constructs were introduced independently into *Agrobacterium tumefaciens* GV3101 and transformed into *A. thaliana* Col-0 using the floral dip method [69]. Flowering times were determined by counting the number of rosette leaves per plant and the days it took for the first flower to bloom.

### Y2H assays

The yeast cloning vectors, pGBKT7 and pGADT7, and the yeast strain AH109 used in the Y2H assays were obtained from Clontech (Mountain View, CA, USA). The *BD-GhFT* vector used in this study was previously published [70]. The coding sequences of *GhFD* and 17 *GhGRF*s were amplified by PCR using gene-specific primers (Additional file 16: Table S8) and then cloned independently into the pMD19-T vector (TaKaRa, Dalian, China) following the manufacturer’s instructions. All of the plasmids were confirmed by sequence analysis, and then *GhFD* and 17 *GhGRF*s were fused to the GAL4 activation domain of pGADT7 to generate *AD-GhFD* and *AD-GhGRF* prey constructs. The Y2H assays were performed in accordance with a method described previously [70].

### BiFC assays

The coding regions of *GhFD* and five *GhGRF*s were amplified using gene-specific primers (Additional file 16: Table S8) and cloned independently into the pDONRZeo vector (Invitrogen) using BP reaction, and then, they were fused to the N-terminus or C-terminus of the PVYNE or PSCYCE vector, respectively [71], using the LR reaction, to generate *nYFP-GhFD* and *cYFP-GhGRF*s. The *nYFP-GhFT* vector used in this study was previously published [70]. The *nYFP-GhFD*, *nYFP-GhFT*, and *cYFP-GhGRF* plasmids were each introduced into *A. tumefaciens* GV3101 cells, which were then infiltrated into *N. benthamiana* leaves for transient expression as described previously [70].

### VIGS assays

For the VIGS assay, each *GhGRF* fragment was cloned by RT-PCR using gene-specific primers (Additional file 16: Table S8) and inserted separately into the *pTRV2* vectors to generate the *pTRV2:GhGRF* constructs. Each *pTRV:GhGRF* plasmid was introduced into *A. tumefaciens* strain GV3101, and infiltrated into cotyledons of *G. hirsutum* L. cv. XLZ 33 as previously described [70, 72].

## Supplementary Information

**Additional file 1: Table S1.** Nomenclature of the *GRF* genes from *G. hirsutum*.

**Additional file 2: Table S2.** Predicted *G. herbaceum*, *G. arboreum*, *G. raimondii*, and *G. hirsutum* GRF proteins with genome identifiers.

**Additional file 3: Table S3.** Features of the *GRF* genes identified from *G. herbaceum*, *G. arboreum*, *G. hirsutum*, and *G. raimondii. (XLSX 14 kb)*

**Additional file 4: Fig. S1.** Chromosomal distributions of the *Gossypium spp*. *GRF* genes. (a) *G. herbaceum*. (b) *G. arboreum*. (c) *G. hirsutum*. (d) *G. raimondii*. Chromosomal locations were shown from top to bottom on corresponding chromosomes according to cotton genome annotation.

**Additional file 5: Fig. S2.** Multiple amino acid sequence alignment of cotton GRF proteins. Amino acid sequence alignment of 82 GRF proteins from *G. herbaceum*, *G. arboreum*, *G. hirsutum*, and *G. raimondii*. Nine α-helices were marked as α1-α9.

**Additional file 6: Table S4.** Seven conserved amino acid motifs and annotation of cotton GRF proteins.

**Additional file 7: Table S5.** Syntenic gene pairs between *G. hirsutum* and other three cotton species.

**Additional file 8: Table S6.** The duplicated *GRF* gene pairs from *G. herbaceum*, *G. arboreum*, *G. hirsutum*, and *G. raimondii*, respectively.

**Additional file 9: Fig. S3.** Distributions of *Ka*/*Ks* values of cotton *GRF* gene pairs. (a) *Ka*/*Ks* ratios for paralogous genes in four cotton species. (b) Boxplot showing the *Ka*/*Ks* ratios for orthologous genes among cotton genomes. The center line in each box indicates the median, and the box limits indicate the upper and lower quartiles of divergence.

**Additional file 10: Fig. S4.** The percentage of various responses about *cis*-acting elements on 2.0-kb promoter of the *GRF* genes in (a) *G. herbaceum*, (b) *G. arboreum*, (c) *G. hirsutum*, and (**d**) *G. raimondii*.

**Additional file 11: Fig. S5.** Information of type, quantity, and location of various response elements in cotton.

**Additional file 12: Table S7.** The FPKM value of *GhGRF*s in different tissues.

**Additional file 13: Fig. S6.** Interaction of GhFT and each GhGRF protein. Plasmids transformed into AH109 yeast strains were screened on the -LT and -LTHA medium.

**Additional file 14: Fig. S7.** Flowering times and the expression levels of the floral-meristem identity genes in the control and *GhGRF* silencing plants. (a–e) Statistics of flowering times in the control and *GhGRF* silencing plants. qRT-PCR analysis of the expression levels of *GhAP1* (Gh_D13G0878) (f–j) and *GhSOC1* (Gh_A11G0755) (k–o). A cotton *Ubiquitin7* (*GhUBQ7*, GenBank accession no. DQ116441) gene was used as an internal reference gene. Values are means ± *SD* (*n* = 3). Asterisks indicate significant differences between control *TRV:00* and *TRV:GhGRF*s silencing lines (Student’s *t* test, *P < 0.05, **P < 0.01, ***P < 0.001, ****P < 0.0001).

**Additional file 15: Fig. S8.** qRT-PCR expression analysis of *GhGRF*, *AtSOC1*, and *AtAP1*. *ACT2* (*At3g18780*) was used as an internal reference transcript. Values are means ± *SD* (n = 3). Asterisks indicate significant differences between Col-0 and the *35S:GhGRF*s transgenic lines (Student’s *t* test, **P < 0.01, ***P < 0.001, ****P < 0.0001).

**Additional file 16: Table S8.** PCR primers used in this study.

## Data Availability

All data generated or analysed during this study are included in this article [and its supplementary information files].

## Abbreviations

GRF: GENERAL REGULATORY FACTOR
FT: FLOWERING LOCUS T
FAC: florigen activation complex
*AP1*: *APETALA1*
*SOC1*: *SUPPRESSOR OF OVEREXPRESSION OF CONSTANS 1*
LD: long-day
GF14s: G-box Factor 14-3-3 homologs
Y2H: yeast two-hybrid
BiFC: bimolecular fluorescence complementation
MeJA: methyl jasmonate
qRT-PCR: quantitative real-time PCR
LT: SD/−Trp-Leu
LTHA: SD/−Trp-Leu-His-Ade
DPA: day post-anthesis
HMM: Hidden Markov Model
FPKM: represented by fragments per kilobase million
*Ka*: non-synonymous
*Ks*: synonymous
VIGS: virus-induced gene silencing
