# Supplementary figures and images for: Roles of the 14-3-3 gene family in cotton flowering
Source: BMC Plant Biol. 2021 Mar 31;21:162. doi: 10.1186/s12870-021-02923-9 (PMC8015177; doi:10.1186/s12870-021-02923-9)

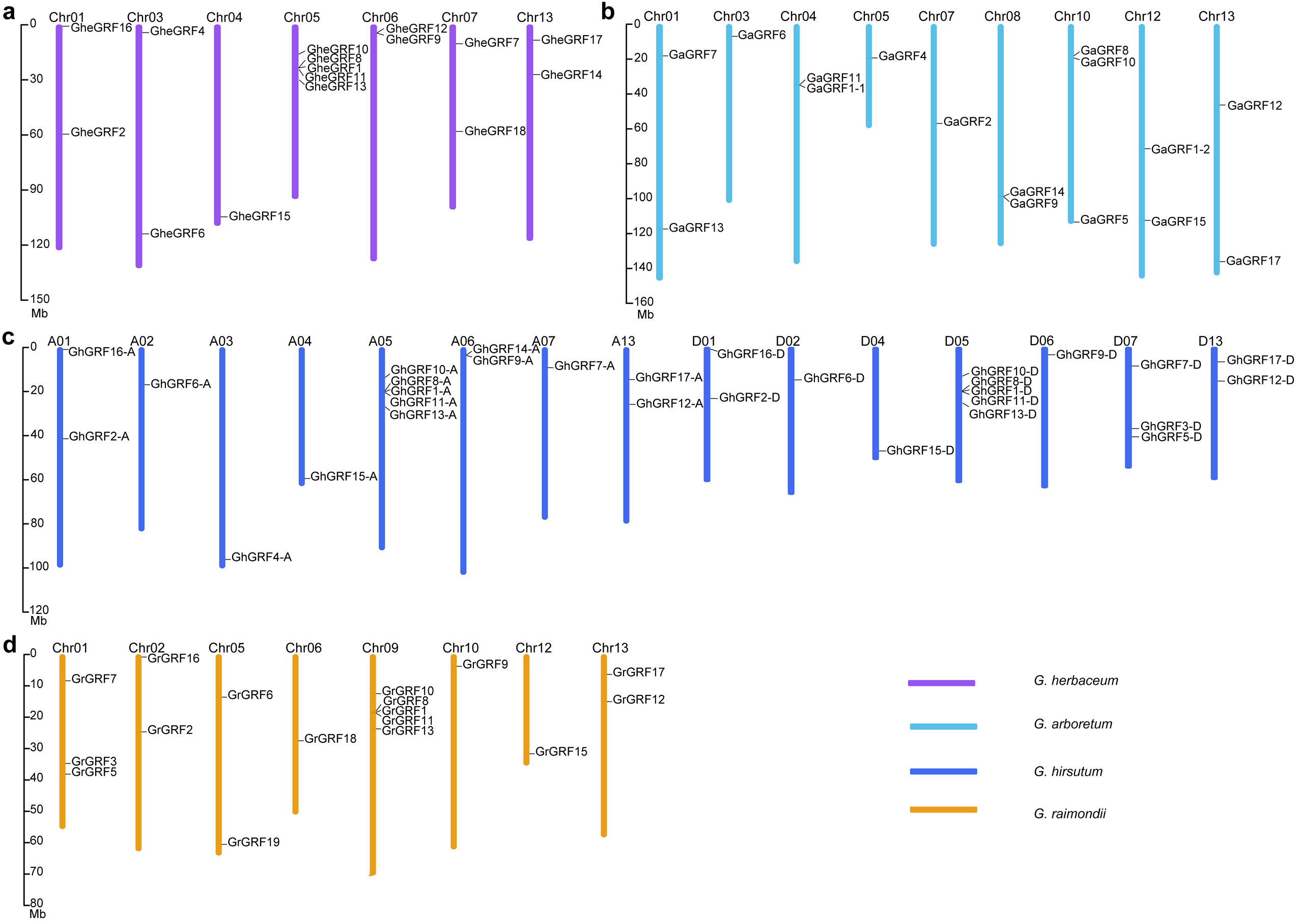

Supplement: Supplementary file 4 — Additional file 4: Fig. S1. Chromosomal distributions of the Gossypium spp. GRF genes. (a) G. herbaceum. (b) G. arboreum. (c) G. hirsutum. (d) G. raimondii. Chromosomal locations were shown from top to bottom on corresponding chromosomes according to cotton genome annotation. [file 12870_2021_2923_MOESM4_ESM.tif]

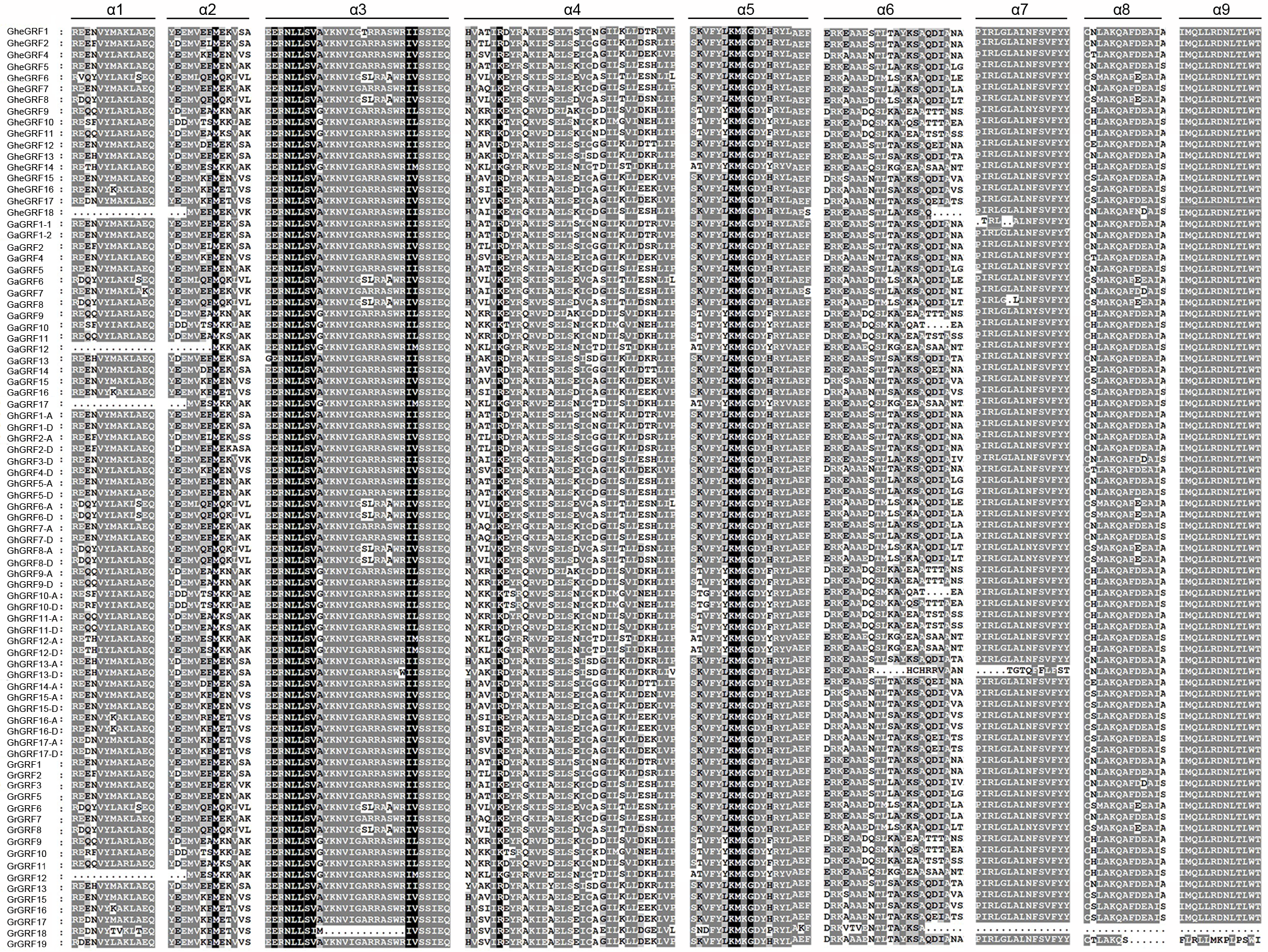

Supplement: Supplementary file 5 — Additional file 5: Fig. S2. Multiple amino acid sequence alignment of cotton GRF proteins. Amino acid sequence alignment of 82 GRF proteins from G. herbaceum, G. arboreum, G. hirsutum, and G. raimondii. Nine α-helices were marked as α1-α9. [file 12870_2021_2923_MOESM5_ESM.tif]

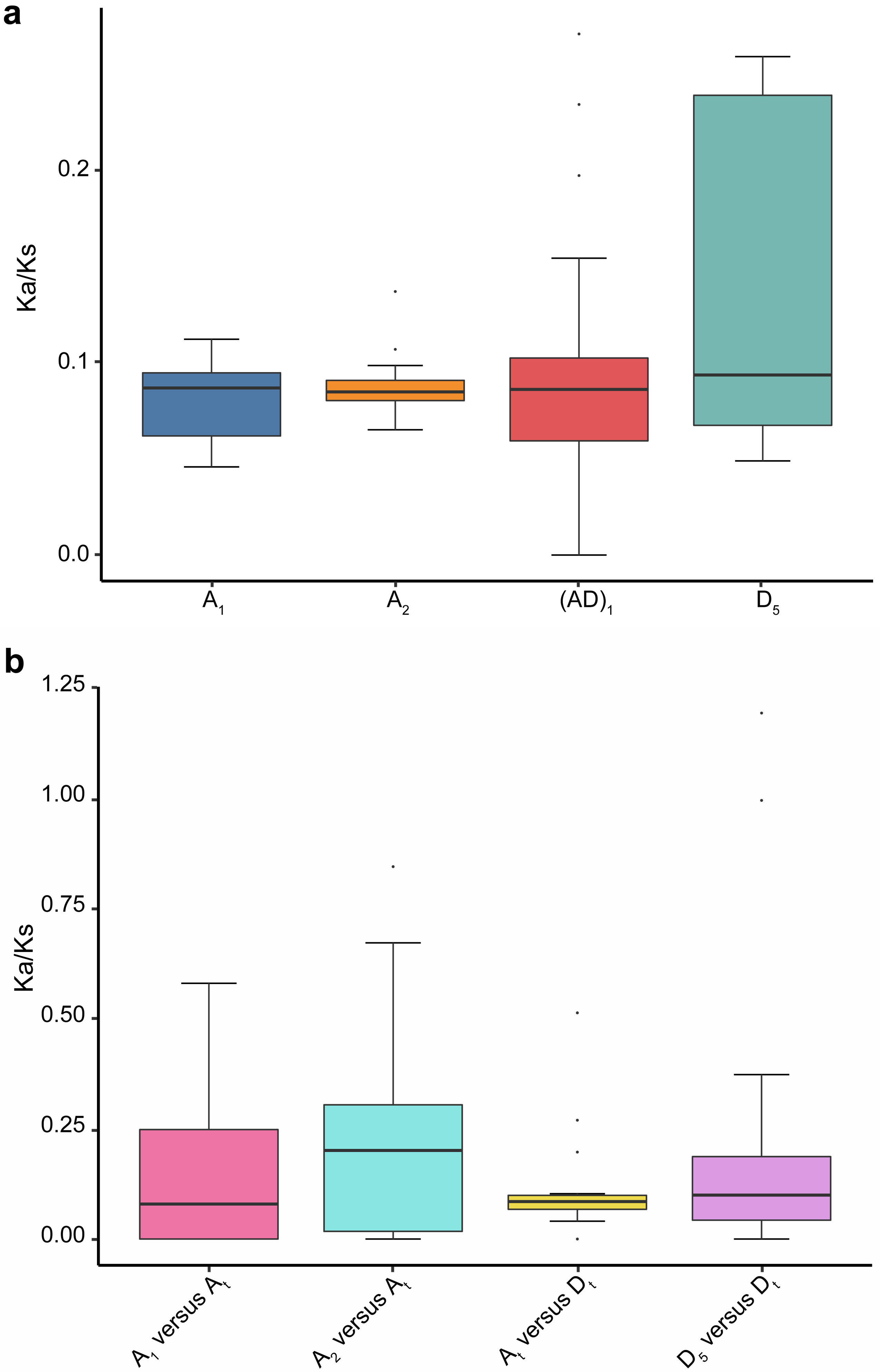

Supplement: Supplementary file 9 — Additional file 9: Fig. S3. Distributions of Ka/Ks values of cotton GRF gene pairs. (a) Ka/Ks ratios for paralogous genes in four cotton species. (b) Boxplot showing the Ka/Ks ratios for orthologous genes among cotton genomes. The center line in each box indicates the median, and the box limits indicate the upper and lower quartiles of divergence. [file 12870_2021_2923_MOESM9_ESM.tif]

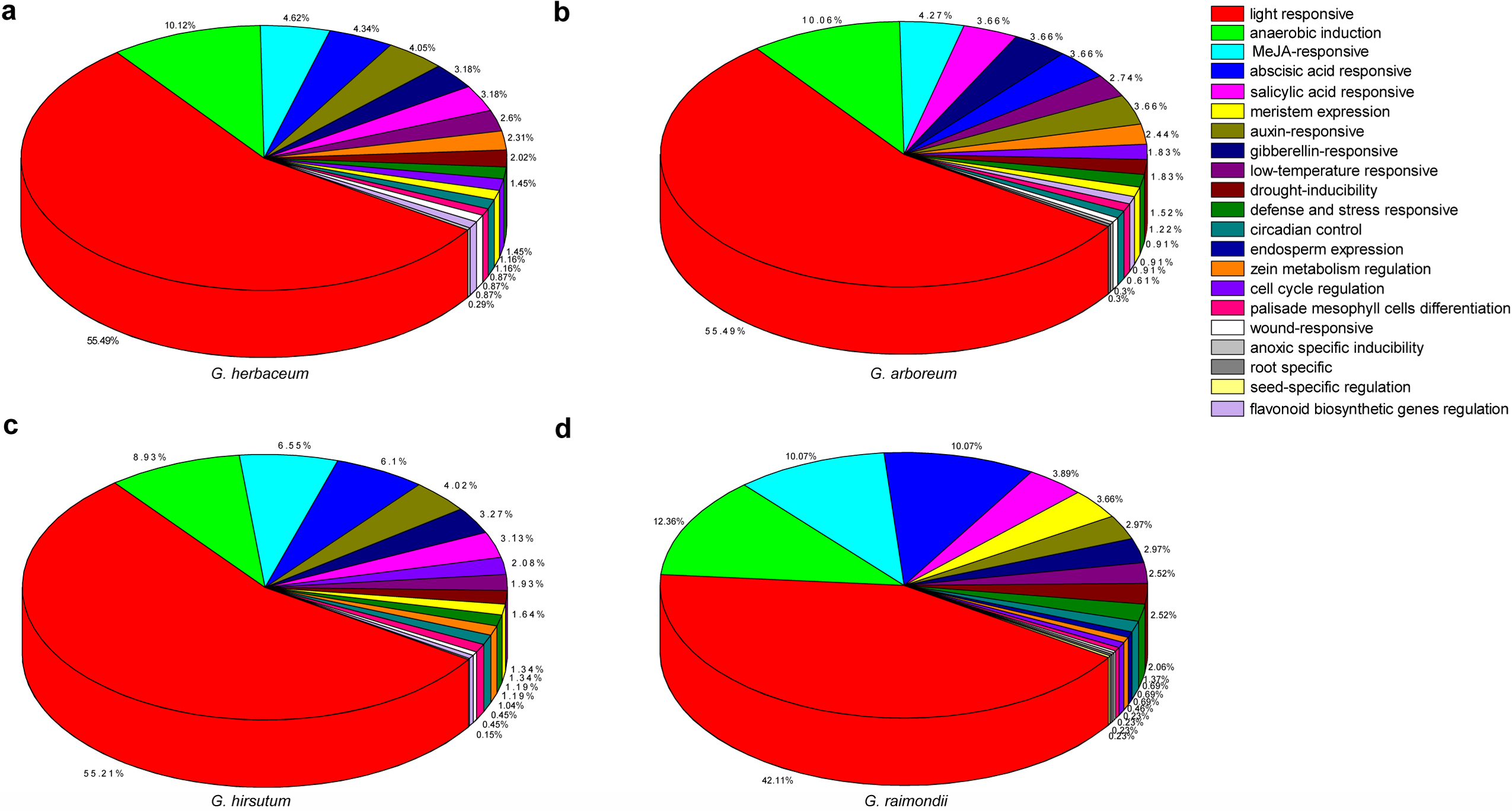

Supplement: Supplementary file 10 — Additional file 10: Fig. S4. The percentage of various responses about cis-acting elements on 2.0-kb promoter of the GRF genes in (a) G. herbaceum, (b) G. arboreum, (c) G. hirsutum, and (d) G. raimondii. [file 12870_2021_2923_MOESM10_ESM.tif]

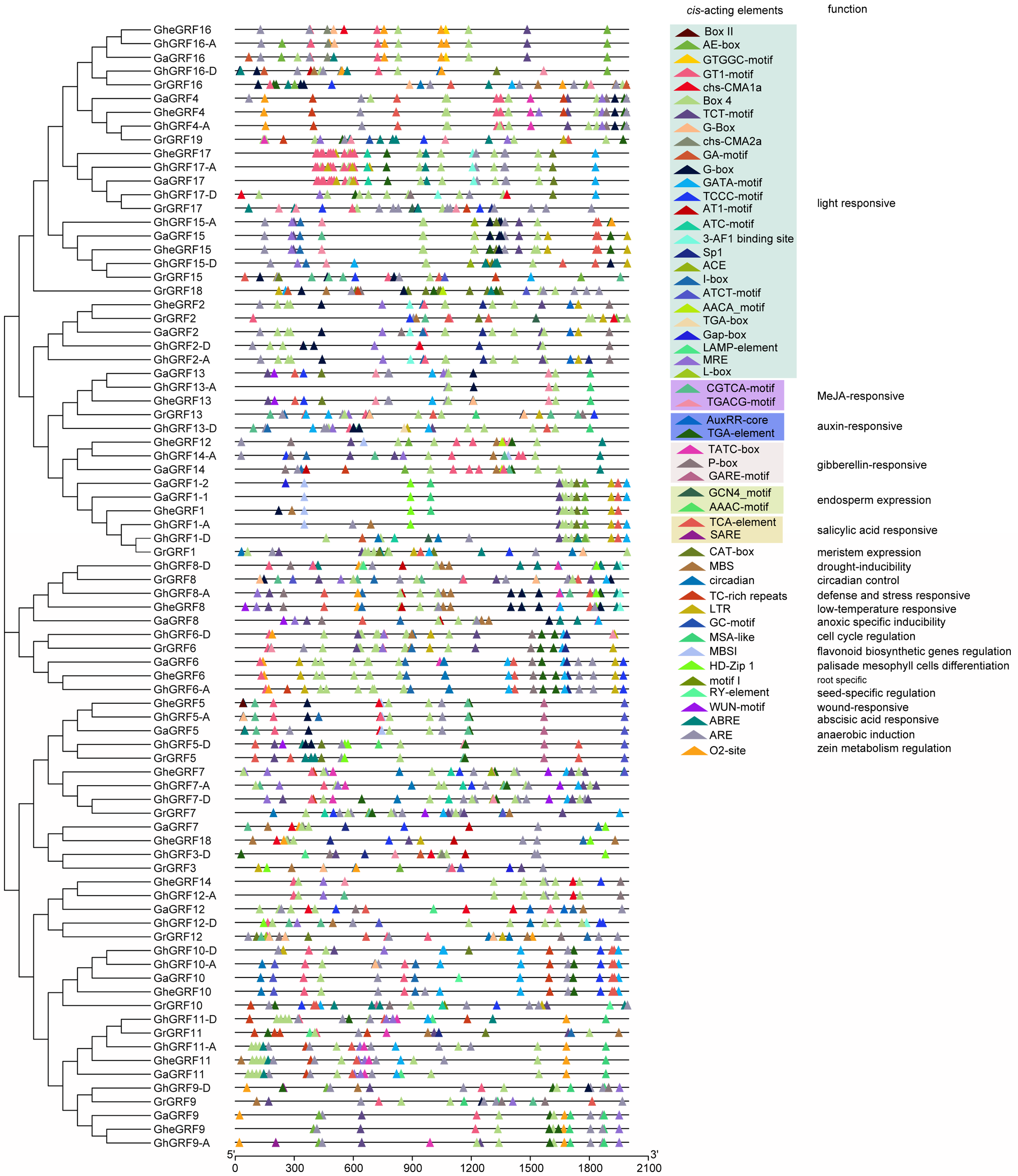

Supplement: Supplementary file 11 — Additional file 11: Fig. S5. Information of type, quantity, and location of various response elements in cotton. [file 12870_2021_2923_MOESM11_ESM.tif]

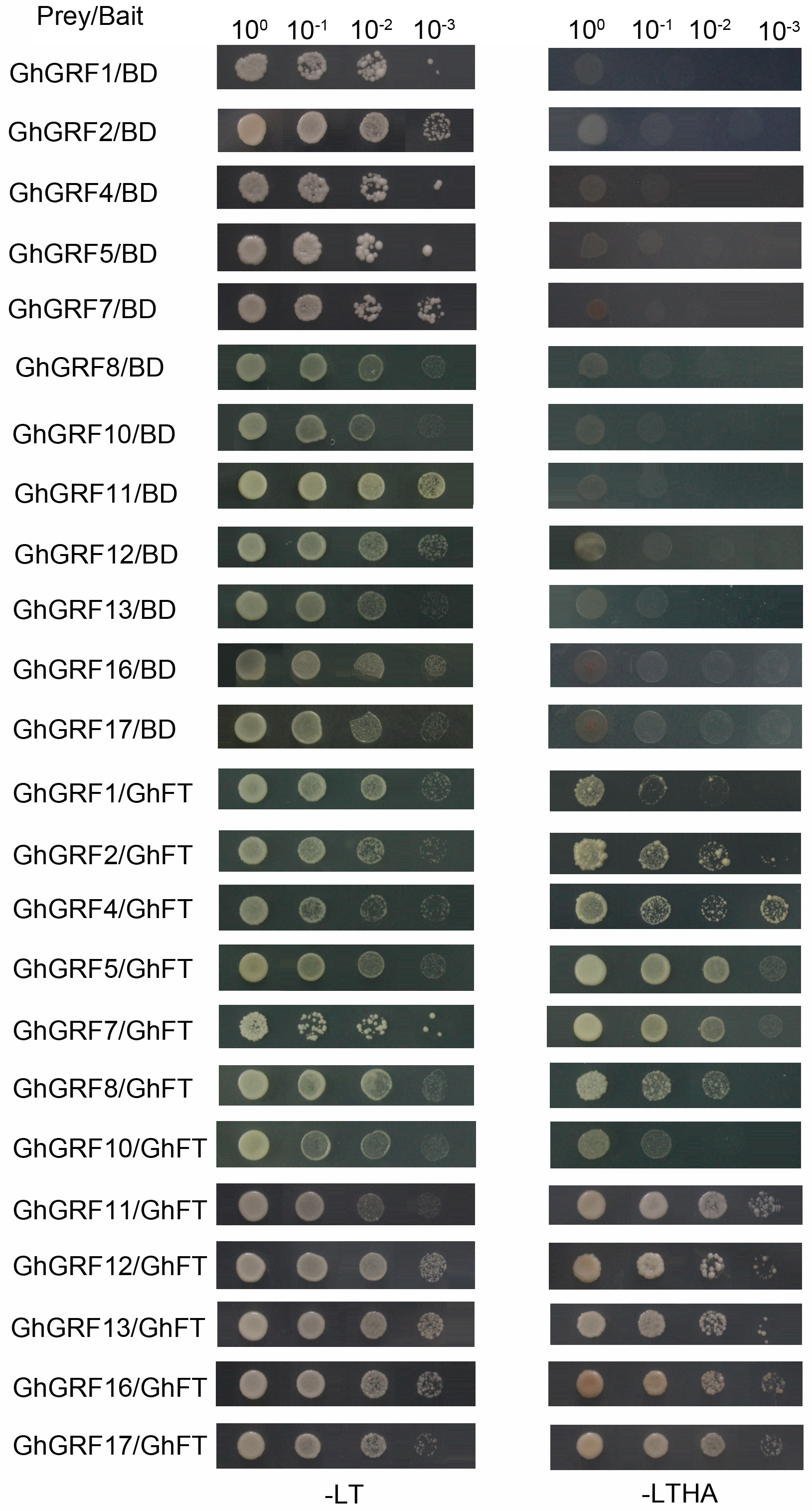

Supplement: Supplementary file 13 — Additional file 13: Fig. S6. Interaction of GhFT and each GhGRF protein. Plasmids transformed into AH109 yeast strains were screened on the -LT and -LTHA medium. [file 12870_2021_2923_MOESM13_ESM.tif]

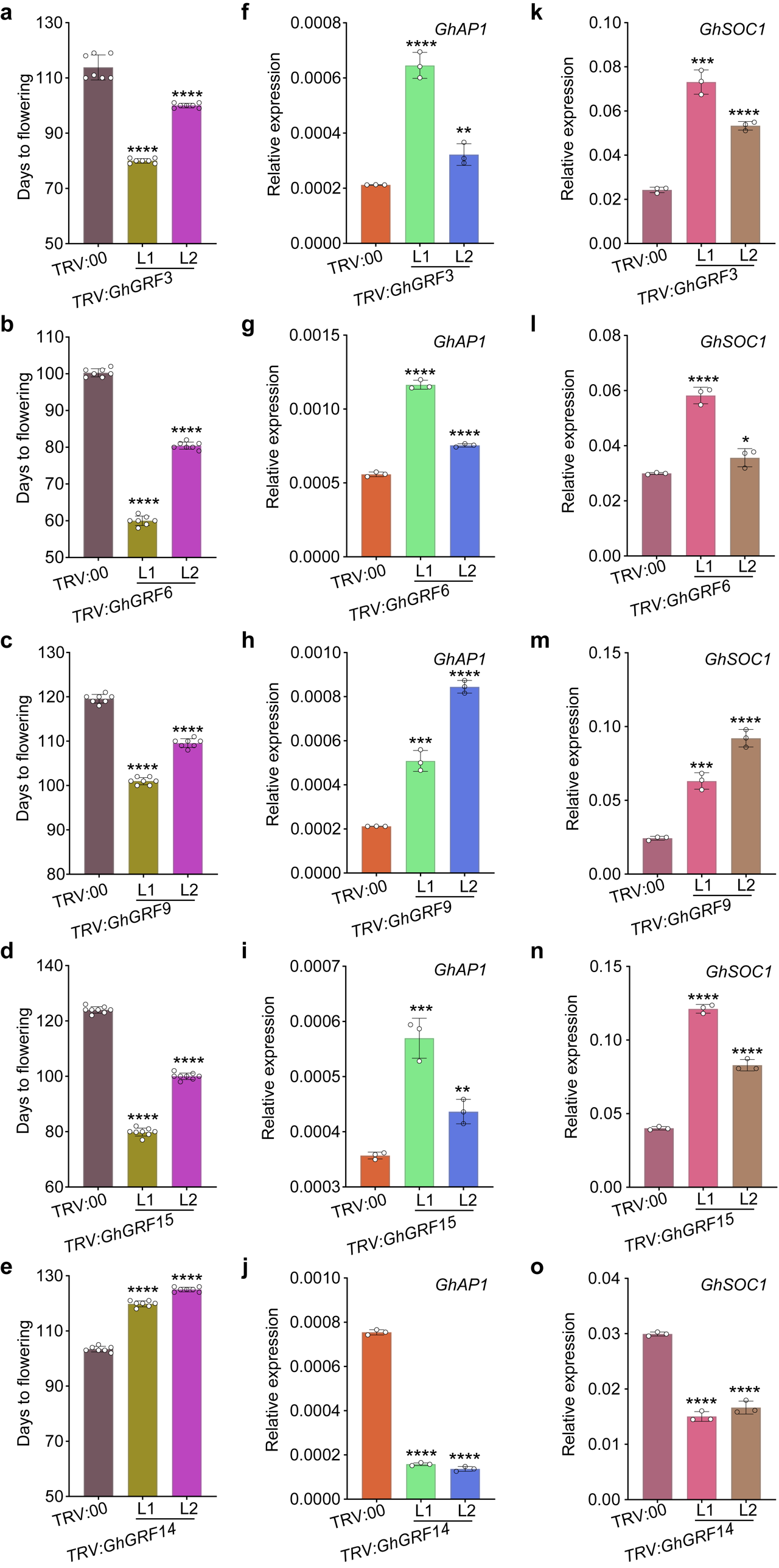

Supplement: Supplementary file 14 — Additional file 14: Fig. S7. Flowering times and the expression levels of the floral-meristem identity genes in the control and GhGRF silencing plants. (a–e) Statistics of flowering times in the control and GhGRF silencing plants. qRT-PCR analysis of the expression levels of GhAP1 (Gh_D13G0878) (f–j) and GhSOC1 (Gh_A11G0755) (k–o). A cotton Ubiquitin7 (GhUBQ7, GenBank accession no. DQ116441) gene was used as an internal reference gene. Values are means ± SD (n = 3). Asterisks indicate significant differences between control TRV:00 and TRV:GhGRFs silencing lines (Student’s t test, *P < 0.05, **P < 0.01, ***P < 0.001, ****P < 0.0001). [file 12870_2021_2923_MOESM14_ESM.tif]

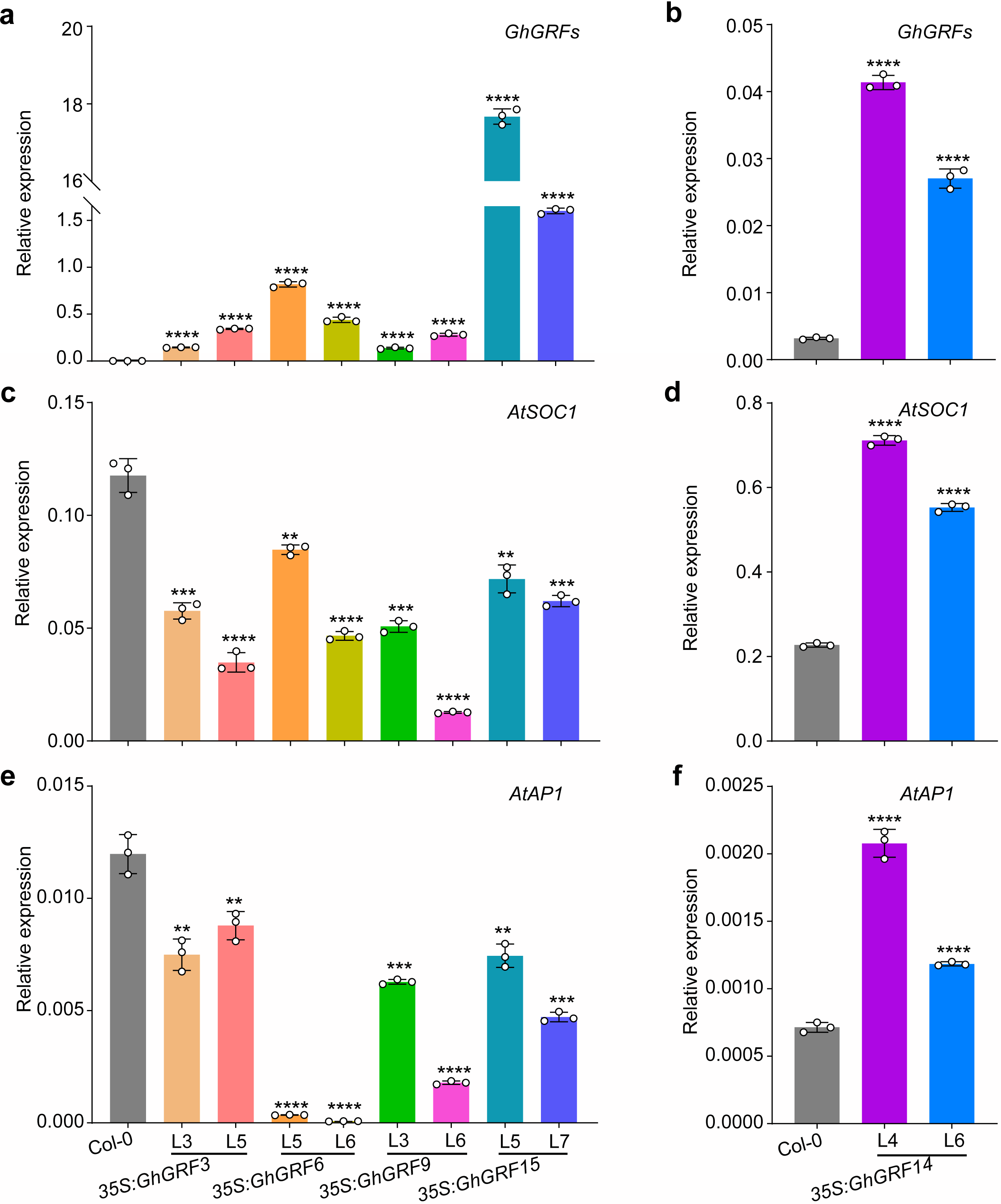

Supplement: Supplementary file 15 — Additional file 15: Fig. S8. qRT-PCR expression analysis of GhGRF, AtSOC1, and AtAP1. ACT2 (At3g18780) was used as an internal reference transcript. Values are means ± SD (n = 3). Asterisks indicate significant differences between Col-0 and the 35S:GhGRFs transgenic lines (Student’s t test, **P < 0.01, ***P < 0.001, ****P < 0.0001). [file 12870_2021_2923_MOESM15_ESM.tif]
